# Chromosome-level genome assembly of *Salvia miltiorrhiza* with orange roots uncovers the role of Sm2OGD3 in catalyzing 15,16-dehydrogenation of tanshinones

**DOI:** 10.1093/hr/uhad069

**Published:** 2023-04-13

**Authors:** Xian Pan, Yujie Chang, Caili Li, Xiaoxiao Qiu, Xinyun Cui, Fanqi Meng, Sixuan Zhang, Xian’en Li, Shanfa Lu

**Affiliations:** Key Lab of Chinese Medicine Resources Conservation, State Administration of Traditional Chinese Medicine of the People' s Republic of China, Institute of Medicinal Plant Development, Chinese Academy of Medical Sciences & Peking Union Medical College, Beijing 100193, China; Engineering Research Center of Chinese Medicine Resource, Ministry of Education, Beijing 100193, China; Key Lab of Chinese Medicine Resources Conservation, State Administration of Traditional Chinese Medicine of the People' s Republic of China, Institute of Medicinal Plant Development, Chinese Academy of Medical Sciences & Peking Union Medical College, Beijing 100193, China; Engineering Research Center of Chinese Medicine Resource, Ministry of Education, Beijing 100193, China; Key Lab of Chinese Medicine Resources Conservation, State Administration of Traditional Chinese Medicine of the People' s Republic of China, Institute of Medicinal Plant Development, Chinese Academy of Medical Sciences & Peking Union Medical College, Beijing 100193, China; Engineering Research Center of Chinese Medicine Resource, Ministry of Education, Beijing 100193, China; Key Lab of Chinese Medicine Resources Conservation, State Administration of Traditional Chinese Medicine of the People' s Republic of China, Institute of Medicinal Plant Development, Chinese Academy of Medical Sciences & Peking Union Medical College, Beijing 100193, China; Engineering Research Center of Chinese Medicine Resource, Ministry of Education, Beijing 100193, China; Key Lab of Chinese Medicine Resources Conservation, State Administration of Traditional Chinese Medicine of the People' s Republic of China, Institute of Medicinal Plant Development, Chinese Academy of Medical Sciences & Peking Union Medical College, Beijing 100193, China; Engineering Research Center of Chinese Medicine Resource, Ministry of Education, Beijing 100193, China; Key Lab of Chinese Medicine Resources Conservation, State Administration of Traditional Chinese Medicine of the People' s Republic of China, Institute of Medicinal Plant Development, Chinese Academy of Medical Sciences & Peking Union Medical College, Beijing 100193, China; Engineering Research Center of Chinese Medicine Resource, Ministry of Education, Beijing 100193, China; Key Lab of Chinese Medicine Resources Conservation, State Administration of Traditional Chinese Medicine of the People' s Republic of China, Institute of Medicinal Plant Development, Chinese Academy of Medical Sciences & Peking Union Medical College, Beijing 100193, China; Engineering Research Center of Chinese Medicine Resource, Ministry of Education, Beijing 100193, China; Key Lab of Chinese Medicine Resources Conservation, State Administration of Traditional Chinese Medicine of the People' s Republic of China, Institute of Medicinal Plant Development, Chinese Academy of Medical Sciences & Peking Union Medical College, Beijing 100193, China; Engineering Research Center of Chinese Medicine Resource, Ministry of Education, Beijing 100193, China; Key Lab of Chinese Medicine Resources Conservation, State Administration of Traditional Chinese Medicine of the People' s Republic of China, Institute of Medicinal Plant Development, Chinese Academy of Medical Sciences & Peking Union Medical College, Beijing 100193, China; Engineering Research Center of Chinese Medicine Resource, Ministry of Education, Beijing 100193, China

## Abstract

*Salvia miltiorrhiza* is well known for its clinical practice in treating heart and cardiovascular diseases. Its roots, used for traditional Chinese medicine materials, are usually brick-red due to accumulation of red pigments, such as tanshinone IIA and tanshinone I. Here we report a *S. miltiorrhiza* line (shh) with orange roots. Compared with the red roots of normal *S. miltiorrhiza* plants, the contents of tanshinones with a single bond at C-15,16 were increased, whereas those with a double bond at C-15,16 were significantly decreased in shh. We assembled a high-quality chromosome-level genome of shh. Phylogenomic analysis showed that the relationship between two *S. miltiorrhiza* lines with red roots was closer than the relationship with shh. It indicates that shh could not be the mutant of an extant *S. miltiorrhiza* line with red roots. Comparative genomic and transcriptomic analyses showed that a 1.0 kb DNA fragment was deleted in shh *Sm2OGD3m*. Complementation assay showed that overexpression of intact *Sm2OGD3* in shh hairy roots recovered furan D-ring tanshinone accumulation. Consistently, *in vitro* protein assay showed that Sm2OGD3 catalyzed the conversion of cyptotanshinone, 15,16-dihydrotanshinone I and 1,2,15,16-tetrahydrotanshinone I into tanshinone IIA, tanshinone I and 1,2-dihydrotanshinone I, respectively. Thus, Sm2OGD3 functions as tanshinone 15,16-dehydrogenase and is a key enzyme in tanshinone biosynthesis. The results provide novel insights into the metabolic network of medicinally important tanshinone compounds.

## Introduction

*Salvia miltiorrhiza* Bunge is one of the most well-known traditional Chinese medicine materials and a model system for medicinal plant biology [1]. It has been clinically used to treat heart and cardiovascular diseases and is beneficial for management of various other diseases, such as cancer and neurodegenerative diseases [2, 3]. Lipophilic tanshinones that are mainly produced in the roots are a major class of bioactive compounds in *S. miltiorrhiza* [4, 5]. Some of them, cryptotanshinone (CT), 15,16-dihydrotanshinone I (15,16-DHT), 1,2,15,16-tetrahydrotanshinone I (THT), and methylenedihydro-tanshinquinone, have a single bond at C-15,16, whereas some of the others, tanshinone I (TAI), 1,2-dihydrotanshinone I (1,2-DHT), tanshinone IIA (TAII), tanshinone IIB, and methylenetanshinquinone, possess a double bond at C-15,16 [6, 7]. Biosynthesis of tanshinones can be generally divided into four stages, including the biosynthesis of C5 isoprene unit isopentenyl diphosphate (IPP) and its isomer dimethylallyl diphosphate (DMAPPP), the formation of intermdediate diphosphate precursor GGPP, the biosynthesis of parent terpene carbon skeletons copalyl diphosphate (CPP) and miltiradiene, and the formation of different tanshinone compounds [1]. So far, the reactions at the first three stages and the genes encoding the enzymes that catalyse these reactions have been elucidated [8–12]. However, due to the complexity, it is still largely unknown about the fourth stage that involves multiple modifications, such as hydroxylation, oxidation, heterocyclization, dehydrogenation, and demethylation.

Recently studies suggest that several cytochromes P450 (CYPs) belonging to the CYP76 and CYP71 subfamilies are involved in tanshinones biosynthesis. SmCYP76AH1 catalyzes the hydroxylation of miltiradiene into ferruginol. Subsequently, ferruginol is further converted to 11-hydroxyferruginol, sugiol and 11-hydroxysugiol under the catalysis of SmCYP76AH3 [13, 14]. The generated 11-hydroxyferruginol and 11-hydroxysugiol are then hydroxylated at C-20 by SmCYP76AK1. SmCYP71D411 catalyzes the hydroxylation of sugiol to 20-hydroxysugiol [14, 15]. In addition, SmCYP71D375 is involved in the hydroxylation and heterocyclization of miltirone, 4-methylenemiltirone and Ro to form the D-ring that has a single bond at C-15,16. Formation of such a D-ring on Ro can also be catalyzed by SmCYP71D373 [15].

In addition to CYP450s, members of the 2-oxoglutarate dependent dioxygenase (2OGD) superfamily have been implied in catalysis of tanshinone biosynthesis [16–18]. 2OGDs are a class of iron-containing non-heme oxygenases localizing in the cytosol and the most versatile oxidative enzymes in nature [19]. They contain the conserved 2-His-1-carboxylate facial triad (HX(D/E)X50–210H) and the conserved RXS motif and catalyze the oxidation of organic substrates using ferrous iron Fe (II) as the active site cofactor and 2-oxoglutarate (2OG) and molecular oxygen as the co-substrates [20]. The reactions they catalyzed include hydroxylation, de-alkylation, demethylation, desaturation, epoxidation, epimerization, cyclization, halogenation, peroxide formation, and ring expansion/contraction [19–23]. The best-studied examples of 2OGDs include GA 20-oxidase, GA 2-oxidase, and GA 3-oxidase that are involved in gibberellin metabolism and flavone synthase I, flavonone 3β-hydroxylase, flavonol synthase and leucoanthocyanidin synthase that are involved in flavonoid metabolism [22, 23]. Compared with CYP450s, 2OGDs are relatively less studied in *S. miltiorrhiza*, although they are often considered as candidate genes involved in tanshinones biosynthesis [16, 24]. *S. miltiorrhiza* has a total of 132 *Sm2OGD* genes [16]. It is reported that down-regulation of *Sm2OGD5* gene expression significantly reduced the contents of miltirone, CT, and TAII in transgenic *S. miltiorrhiza* hairy roots [16]. The reaction catalyzed by 2OGD5 protein is unknown. In addition, it has been shown that Sm2OGD25 catalyzes the hydroxylation of sugiol to produce hypargenin B and crossogumerin C [17]. Sm2-ODD14 converts CT and isocryptotanshinone to TAII and isotanshione IIA, respectively [18].

The roots and rhizomes of *S. miltiorrhiza* have been used as traditional Chinese medicine materials for a long time. They are called Danshen and mean ‘red root’ in Chinese due to the substantial accumulation of red tanshinones, such as TAII and TAI in root periderm [4, 5, 25, 26]. In this study, we investigated a *S. miltiorrhiza* line shh that has orange roots. In comparison with tanshinone contents in red roots of normal *S. miltiorrhiza* plants, the contents of tanshinones with a single bond at C-15,16 were increased in orange roots, whereas the contents of those unsaturated at C-15 and C-16 were significantly decreased. In order to obtain more comprehensive information to explain the large variations of tanshinone contents in shh, we sequenced the whole genome and assembled it to the chromosome level. The comparative genomic and transcriptomic analyses showed that the *Sm2OGD3* gene was mutated by fragment deletion in shh. Further enzyme activity analysis and transgenic hairy roots experiment of intact Sm2OGD3 protein revealed its function in catalyzing the dehydrogenation of tanshinones at C-15 and C-16. Our results suggest that fragment deletion in *Sm2OGD3* gene in shh could be responsible for the lack of 15,16-unsaturated tanshinones.

## Results

### Comparative metabolome and UPLC analysis of tanshinones in *S. miltiorrhiza* lines with orange roots (shh) and red roots (99–3)

**Figure 1:**
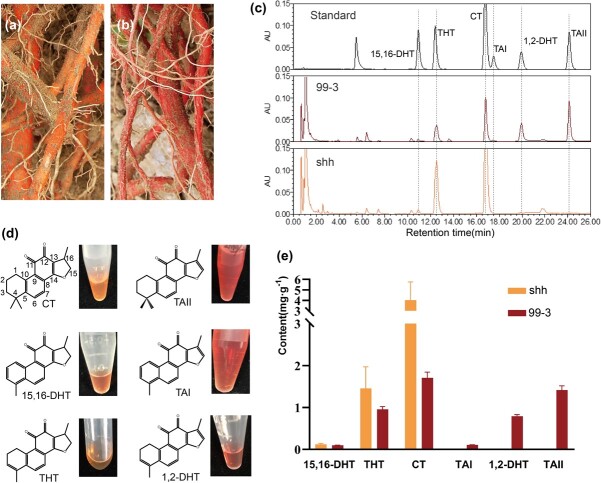
UPLC analysis of tanshinones in *Salvia miltiorrhiza* lines shh and 99–3. Different color phenotypes of shh (**a**) with orange roots and 99–3 (**b**) with red roots. **c** UPLC analysis of mature roots of 99–3 and shh. **d** Color characteristic of tanshinone compounds. The concentration of CT and TAII were 2 mg.ml^−1^. 15,16-DHT and TAI were at a concentration of 0.5 mg.ml^−1^. THT and 1,2-DHT were at a concentration of 1 mg.ml^−1^. **e** The contents of tanshinone compounds in the mature roots of shh and 99–3. The error bars denote 1SE. CT, cryptotanshinone; TAII, tanshinone IIA; 15,16-DHT, 15,16-dihydrotanshinone I; TAI, tanshinone I; THT, 1,2,15,16-tetrahydrotanshinone I; 1,2-DHT, 1,2-dihydrotanshinone I.

Tanshinones are enriched in the periderm of *S. miltiorrhiza* roots [4, 5]. Many tanshinone compounds show colors, ranging from orange to red [25, 26]. For instance, the methanolic solution of TAII, TAI, and 1,2-DHT are red, whereas CT, 15,16-DHT, and THT are orange (Fig. 1d). Normal *S. miltiorrhiza* plants, such as line 99–3, are brick-red, because of the accumulation of TAII, TAI, 1,2-DHT, and other red tanshinones (Fig. 1b). However, the roots of a nature line of *S. miltiorrhiza* were orange (Fig. 1a). This line was originally found in Shaanxi in China, in 1999. It has been transplanted to the field nursery of the Institute of Medicinal Plant Development of the Chinese Academy of Medical Sciences and propagated through root cuttings for about 20 years. This line was named Shanhuang (shh).

In order to analyse the changes of chemical compositions in shh, we performed comparative metabolomic analysis of roots from lines 99–3 and shh. The content of metabolites was evaluated using OrthogonalPartial Least Squares Discrimination Analysis (OPLS-DA) with the threshold for significant differences determined by variable importance in the projection (VIP) >1.0, *P*-value <0.05, and fold change >1.5 or <0.67. It resulted in the identification of 29 compounds with contents significantly changed in shh, among which TAII, CT, THT, and TAI were the four compounds with the largest VIP (Table S1, see online supplementary material). TAII, TAI, and methyl tanshinoate that possess a double bond at C-15,16 were decreased to 17.78-, 46.24-, and 39.06-folds, respectively. Tanshinones with a single bond at C-15,16, such as THT, CT and its derivatives, cryptomethyltanshinoate and anhydride of CT, were increased to 2.24-, 1.76-, 3.87-, and 19.76-folds, respectively. In addition, the upstream products of the tanshinone biosynthetic pathway, such as sugiol, miltirone I and dehydromiltirone, were increased to 2.00-, 1.94-, and 2.56-folds, respectively.

Significant variations of tanshinone production between shh and 99–3 were further confirmed using ultra-high-performance liquid chromatography (UPLC) (Fig. 1c). In 99–3 roots, the contents of TAII, TAI, and 1,2-DHT with a double bond at C-15,16 were 1.42, 0.11, and 0.79 mg g^−1^ fresh weight, respectively, whereas they were almost undetectable in shh roots (Fig. 1e). On the contrary, CT, 15,16-DHT, and THT with a single bond at C-15,16 were increased to 2.36, 1.23, and 1.52 folds in shh roots, respectively, when compared with those in 99–3 roots (Fig. 1e). Taken together, we proposed that 15,16-dehydrogenation was impaired in shh.

### Chromosome-level genome assembly of *S. miltiorrhiza* line with orange roots (shh)

**Figure 2:**
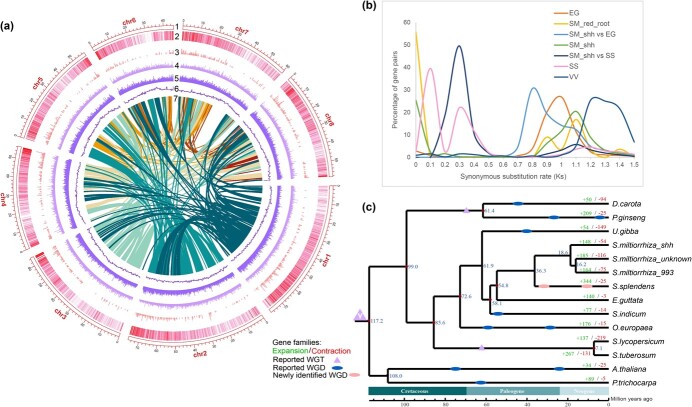
Genome features, *K*s distributions and phylogenomic analysis of *Salvia miltiorrhiza* line shh. **a** Overview of shh genome assembly. The outer layer of hollow blocks represents the eight seudochromosomes (1). Tracks (2) to (5) represent density of gene, differential expressed gene between young root and mature roots of shh, long terminal repeats, and transposable elements, respectively. Tracks (6) and (7) show GC content and syntenic blocks, respectively. **b** The synonymous substitution rate (*K*s) distributions of *Erythranthe guttata* (EG) paralogs, *S. miltiorrhiza* with red roots (SM_red_root) paralogs, *S. miltiorrhiza* line shh (SM_shh) paralogs, *Salvia splendens* (SS) paralogs and *Vitis vinifera* (VV) paralogs and the *K*s distributions of homologous gene pairs in the collinearity block of SM_shh vs SS, SM_shh vs EG. **c** Phylogenetic tree of shh and other 13 species, including three *S. miltiorrhiza* line 99–3, *S. miltiorrhiza* line unknown, *S. splendens*, *Erythranthe guttata*, *Sesamum indicum*, *Utricularia gibba*, *Olea europaea*, *Solanum lycopersicum*, *Solanum tuberosum*, *Panax ginseng*, *Daucus carota*, *Arabidopsis thaliana*, and *Populus trichocarpa*.

So far, the whole genome assemblies have been available for four *S. miltiorrhiza* lines with red roots [15, 27–29]. However, there is no genomic sequence available for *S. miltiorrhiza* plants with orange roots. In order to investigate the genes responsible for the variations in tanshinone production in shh, we sequenced and assembled the whole genome of shh to chromosome level (Fig. 2a). The *K*-mer 17 distribution analysis of 28.73 Gb Illumina short reads estimated that the size of shh genome was 581.68 Mb and the heterozygosity is 1.34% (Table S2, see online supplementary material). The genome was sequenced through a combination of different sequencing technologies (Table S3, see online supplementary material). It resulted in the acquirement of 64.90 Gb PacBio long reads, 66.57 Gb Illumina reads, and 124.14 Gb 10× genomics barcoded reads. The initial genome assembly obtained had a total of 530.97 Mb with a scaffold N50 size of 2.01 Mb and contig N50 of 1.05 Mb (Table 1). It covered 91.28% of the genome size estimated by genome survey. The contig N50 is approximately 2- to 83-fold longer than the previous assemblies of the *S. miltiorrhiza* genomes [15, 27–29] (Table 2). Furthermore, 133.51 Gb Hi-C data were used to assist the assembly [30]. A total of 371 scaffolds covering 496.5 Mb (93.51%) of the assembled genome were anchored into eight pseudochromosomes (Figs S1 and S2, see online supplementary material). A super-scaffold N50 of 60.28 Mb and a maximum scaffold length of 77.73 Mb were attained after Hi-C (Table 1; Table S4, see online supplementary material).

To assess the completeness of the assembly and the homogeneity of the sequencing, Illumina short reads were mapped to the genome using BWA [31]. It exhibits exceptional alignments with the mapping rate of 95.96%, the coverage of 99.74%, and the average sequence depth of 46.77%. BUSCO [32] and CEGMA [33] assessments were also performed, resulting in the identification of 88% single-copy orthologs and 89.52% core eukaryotic conserved genes, respectively (Table S5, see online supplementary material). It indicates that the chromosome-level genome assembly of *S. miltiorrhiza* line shh has a high degree of continuity and completeness.

Whole genome repeats were identified by homology alignment and *de novo* search (Tables S6 and S7, see online supplementary material). The results showed that shh genome contained about 56.65% repetitive sequences (Fig. 2a). Long terminal repeats (LTRs) were the predominant transposable elements, which represented 49.30% of the whole genome. Based on *ab initio* prediction, homology-based prediction and RNA-Seq assisted prediction, a total of 32 191 protein-coding genes with an average CDS length of 1191 bp were predicted (Table 1; Table S8, see online supplementary material). The number of predicted gene models was similar to previously reported *S. miltiorrhiza* genomes [15, 27–29] (Table 2). Among the 32 191 predicted protein-coding genes, 30 538 (94.9%) were functionally annotated by mapping to protein databeses, including Swiss-Prot, NR, KEGG, InterPro, GO, and Pfam (Table S9, see online supplementary material).

### Phylogenomic analysis revealed the evolutionary history of the orange root line (shh)

With the whole genome assembly available, we asked how shh was evolved. To address this question, shh genome assembly was compared with the whole genomes from the other plant species, including five *Salvia* species (*S. miltiorrhiza*_99–3, *S. miltiorrhiza*_unknown, *S. miltiorrhiza*_DSS3, *Salvia bowleyana*, *Salvia hispanica, Salvia splendens*), seven Lamiales species (*Erythranthe guttata*, *Sesamum indicum*, *Utricularia gibba*, *Olea europaea*, *Scutellaria baicalensis*), two Solanaceae species (*Solanum lycopersicum*, *Solanum tuberosum*), two Apiales species (*Panax ginseng*, *Daucus carota*), *Arabidopsis thaliana*, and *Populus trichocarpa*. The phylogenomic tree was constructed based on single-copy gene families using maximum likelihood estimation.

**Table 1:** Statistics and characteristics of shh genome.

**Parameter**	**Value**
Estimated genome size (by *k*-mer analysis)	581.68 Mb
Assembly size	530.97 Mb
Contig N50	1.05 Mb
Scaffold N50	2.01 Mb
Scaffold N50 (after Hi-C)	60.28 Mb
Longest scaffold	77.73 Mb
Chromosome anchoring rate	94.9%
Number of protein-coding genes	32 191
Average transcript length	3034 bp
Average CDS length	1191 bp

**Table 2:** Comparison of shh assembly with other four published *Salvia miltiorrhiza* genome assemblies.

**Line**	**Genome size**	**Sequencing strategy**	**Contig/Scaffold N50**	**Assembly level**	**Completeness**	**No. of annotated protein coding genes**
Unknown	641 Mb	Illumina PacBio RS II	82.8 Kb/1.2 Mb	Scaffold	89.11%	34 598
99–3	538 Mb	Illumina PacBio RS II Roche 454	12.38 Kb/51.02 Kb	Scaffold	NA	30 478
DSS3	594.75 Mb	PacBio Sequel Hi-C	2.7 Mb/NA	Chromosome	92.5%	32 483
bh2–7	557 Mb	Illumina PacBio RS	505.21 Kb/1.26 Mb	Scaffold	91.10%	33 760
shh	530.97 Mb	PacBio Sequel Illumina 10X Genomics Hi-C	1.01 Mb/2.01 Mb	Chromosome	89.52%	32 191

NA, not available; PacBio, Pacific Biosciences Single Molecule Real-Time technology; Hi-C, high-throughput/resolution chromosome conformation capture.

The phylogenomic tree showed that three *S. miltiorrhiza* lines were clustered with *S. splendens*, whereas *S. splendens* diverged from the common ancestor of *S. miltiorrhiza* approximately 36.3 million years ago (MYA) (Fig. 2c). Interestingly, two *S. miltiorrhiza* lines having red roots showed a close relationship with the divergence time about 16.2 MYA, whereas the divergence of shh from the common ancestor of the *S. miltiorrhiza* lines with red roots occurred about 18.6 MYA (Fig. 2c). However, when we added *S. miltiorrhiza*_DSS3, *S. bowleyana*, *S. hispanica* and *S. baicalensis* to the new phylogenetic tree, *S. bowleyana* was clustered with several *S. miltiorrhiza* lines and even showed the closest relationship with *S. miltiorrhiza* 99–3 (Fig. S3, see online supplementary material). *S. bowleyana* and *S. miltiorrhiza* line 99–3 diverged approximately 10.4 MYA (Fig. S4, see online supplementary material). In a previous study, *S. bowleyana* and *S. miltiorrhiza* diverged within the Lamiaceae branch approximately 3.94 MYA [34]. This indicates that the two plants are very similar at the molecular level. The roots of *S. bowleyana* have been reported to be used as a substitute for *S. miltiorrhiza* [34]. The divergence of shh from the common ancestor of *S. miltiorrhiza* 99–3 and *S. bowleyana* occurred about 13.8 MYA (Fig. S4, see online supplementary material). *S. miltiorrhiza* line shh with orange roots was clustered with *S. miltiorrhiza* lines with red roots and *S. bowleyana. *It indicates that shh could not be the mutant of an extant *S. miltiorrhiza* line with red roots. The common ancestor of shh, *S. miltiorrhiza* with red roots and *S. bowleyana* may be a red-rooted species.

We next asked whether there was a lineage-specific whole-genome duplication (WGD) event occurring in *S. miltiorrhiza* plants with orange roots and this event resulted in the changes of tanshinone production. To address this question, distribution of synonymous substitutions per synonymous site (*K*s) analysis for paralogous genes and syntenic blocks was carried out. The results showed that *S. splendens* experienced twice lineage-specific WGD events after the divergence from *S. miltiorrhiza*, whereas there was no recent WGD event occurring in shh (Fig. 2b). Lack of recent WGD events was also found in *S. miltiorrhiza* lines with red roots (Fig. 2b). It indicated that no recent WGD could be a common phenomenon for all *S. miltiorrhiza* plants and the changes of tanshinone production in shh were not caused by WGD events.

### Comparative transcriptomic analysis of known genes involved in tanshinone biosynthesis in orange roots (shh) and red roots (99–3) of *S. miltiorrhiza* lines

**Figure 3:**
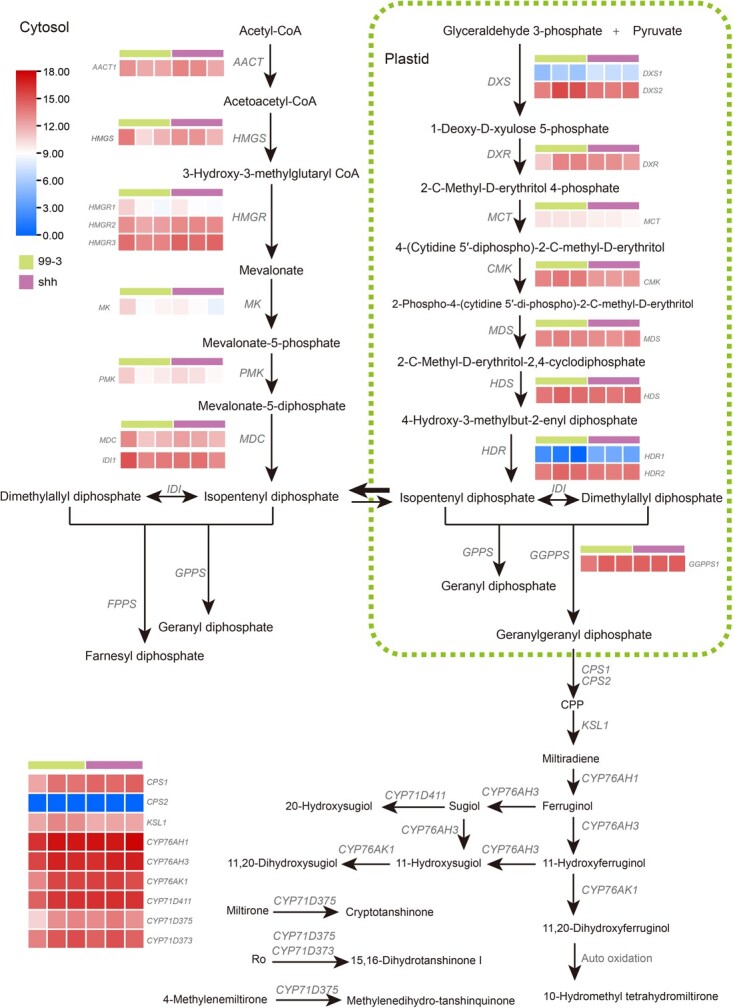
RNA-seq analysis of known genes involved in tanshinone biosynthesis in the periderm of mature roots of shh and 99–3. Three biological replicates of each line were applied. TPM with log scale disposed by ZeroToOne normalization was used.

Tanshinones are a gathering of lipophilic diterpenoid natural products biosynthesized through the terpenoid biosynthetic pathway. So far, a total of 19 gene families have been found to encode enzymes associated with tanshinones production in the *S. miltiorrhiza* plants with red roots [1]. To get the first-hand information for these genes in shh, we performed genome-wide identification of shh homologs for those known enzyme-encoding genes [8, 9, 11–13, 15]. Homologs for all of the known enzyme-encoding genes implicated in tanshinone production could be found in the genome assembly of shh (Table S10, see online supplementary material). Comparative transcriptomic analysis showed that there was no significant difference for their expression in the periderm of mature roots of shh and 99–3 (Fig. 3). The results suggested that the variations of tanshinone production in shh were not caused by loss-of-function or significant expression level changes of those known enzyme-encoding genes.

### Comparative genomic and transcriptomic analyses showed that *Sm2OGD3* gene was mutated by fragment deletion in the orange root line (shh)

**Figure 4:**
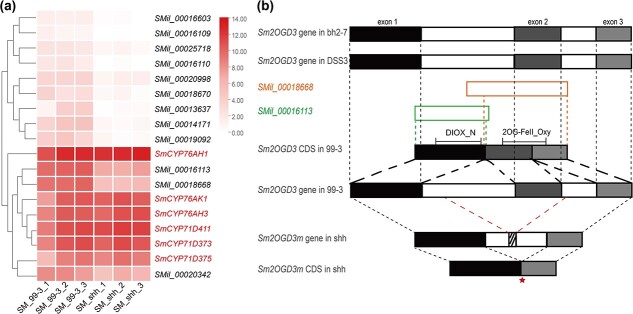
Genomic and transcriptomic analyses of *Sm2OGD3* gene in *Salvia miltiorrhiza*. **a** Expression level of 12 differentially expressed *Sm2OGDs* in the mature periderm of shh and 99–3 visualized by heatmap. Cluster analysis shows *SMil_00016113*, *SMil_00018668*, and *SMil_00020342* presented similar expression profiles with SmCYP76AH1, SmCYP76AH3, SmCYP76AK1, SmCYP71D411, SmCYP71D373, and SmCYP71D375, which are already known functions in tanshinone biosynthesis. **b** Comparative analysis of *Sm2OGD3* gene in *S. miltiorrhiza* lines bh2–7, DSS3, 99–3, and shh. *Sm2OGD3* gene has three exons (filled boxes) and two introns (white boxes with black frame). Two gene models, *SMil_00018668* and *SMil_00016113* predicted from 99–3 genome assembly are represented by white boxes with orange and green frames, respectively. Two conserved domains of Sm2OGD3 protein, DIOX_N and 2OG-FeII_Oxy, are shown. The red dotted line indicates that *Sm2OGD3m* gene loses a 1.0 kb DNA fragment containing the entire second exon and partial of the first and the second intron sequences. The red asterisk indicates the first stop codon.

To further investigate the reasons for the variations of tanshinone contents in shh, comparative transcriptomic analysis was performed using our RNA-seq data from mature periderm of 99–3 and shh roots [24]. A total of 3416 differentially expressed genes (DEGs) with a significance level of padj <0.05 were identified between 99–3 and shh root periderm (Fig. S5, see online supplementary material). Among them, 2162 DEGs were down-regulated in shh. It includes 12 *Sm2OGD* gene models (Fig. 4a). 2OGDs are a class of oxygenases encoding by a gene superfamily. Members of the *S. miltiorrhiza 2OGD* gene family have been recently implied in the catalysis of tanshinone biosynthesis [16–18, 24]. In order to check whether the *Sm2OGDs* were involved in the production of tanshinones with a double bond at C-15,16, we screened candidate *Sm2OGD* genes based on the following criteria, including (i) significantly down-regulated in shh periderm in comparison with 99–3, (i) highly expressed in 99–3 periderm, and (iii) specifically highly expressed in 99–3 roots among different organs (flower, stem, leaf, and root). As shown in the clustering heatmap, among the 12 *Sm2OGD* gene models, SMil_00016113, SMil_00018668, and SMil_00020342 co-expressed with SmCYP76AH1, SmCYP76AH3, SmCYP76AK1, SmCYP71D411, SmCYP71D373, and SmCYP71D375 that were involved in tanshinone biosynthesis (Fig. 4a; Fig. S6, see online supplementary material). Based on the previously published article [16], *SMil_00020342* was expressed much higher in stems than roots of 99–3 (Fig. S7, see online supplementary material), indicating it was not related to tanshinone biosynthesis. Thus, *SMil_00016113* and *SMil_00018668* were selected for further analysis.

*SMil_00018668* was named *Sm2OGD3* in a previously published article [16]. However, BLASTp analysis against the NCBI NR database showed that the proteins encoded by *SMil_00016113* and *SMil_00018668* were partial. *SMil_00016113*, which includes a DIOX_N domain, was mapped to the 5′ region of a 2OGD. *SMil_00018668* that includes a 2OG-FeII_Oxy domain was mapped to the 3′ region (Fig. 4b). To address this problem, we performed BLASTn analysis of *SMil_00016113* and *SMil_00018668* against the genome assemblies of three *S. miltiorrhiza* lines with red roots, including 99–3 [28], bh2–7 [15], and DSS3 [29]. *SMil_00016113* and *SMil_00018668* were mapped to a *Sm2OGD* gene with three exons in the genome assemblies of bh2–7 [15] and DSS3 [29]. *SMil_00016113* corresponds to the first exon, whereas *SMil_00018668* corresponds to the second and third exons (Fig. 4b). When mapping *SMil_00016113* and *SMil_00018668* to the genome assembly of 99–3 [28], we found that *SMil_00016113* and *SMil_00018668* were located at scaffold5164 and scaffold2881, respectively. Because the quality of 99–3 genome assembly was relatively low, it is possible that the sequences of *SMil_00016113* and *SMil_00018668* were incorrectly assembled. To exam whether *SMil_00016113* and *SMil_00018668* also came from a *Sm2OGD* gene in 99–3, primers were designed (Table S11, see online supplementary material) and PCR amplification were carried out using gDNA and cDNA from 99–3 roots as the templates. The results showed that *SMil_00016113* and *SMil_00018668* indeed came from a *Sm2OGD* gene in 99–3 (Fig. 4b). Based on these results, we corrected the full-length genomic sequence and CDS of *Sm2OGD3* in 99–3 (Table S12, see online supplementary material). The corrected CDS has an open reading frame of 1119 nt and encodes a protein with 373 amino acids.

BLASTn analysis of *SMil_00016113* and *SMil_00018668* against the genome assembly of shh identified a 1.2 kb genomic sequence. Its 5′ region corresponds to *SMil_00016113*, its 3′ region corresponds to the 3′ region of *SMil_00018668*, whereas the genomic region corresponding to the 5′ region of *SMil_00018668* is missing (Fig. 4b). Compared to the genomic sequence of *Sm2OGD3* in 99–3, the identified 1.2 kb genomic sequence of shh lacked a DNA fragment of approximately 1.0 kb in length, which corresponds to the second exon and partial of the first and the second intron sequences (Fig. 4b). We named this mutated gene in shh as *Sm2OGD3m*. Again, we performed PCR amplification using gDNA and cDNA templates from shh roots and the same primers. According to the full-length genomic sequence and CDS of *Sm2OGD3m* in shh (Table S12, see online supplementary material), we found that *Sm2OGD3m* underwent an exon skip type of variable splicing due to a fragment deletion that disrupted the original splice site, with only the first and third exons remaining intact in the CDS. However, the misalignment of the reading frame resulted in the premature termination of translation. The first stop codon occurs at 541-543 nt and the truncated protein is only 180aa. It results in deprivation of the important conserved 2-His-1-carboxylate facial triad and the conserved RXS motif [20].

### Sm2OGD3 recombinant protein catalyzed 15,16-dehydrogenation of tanshinones

**Figure 5:**
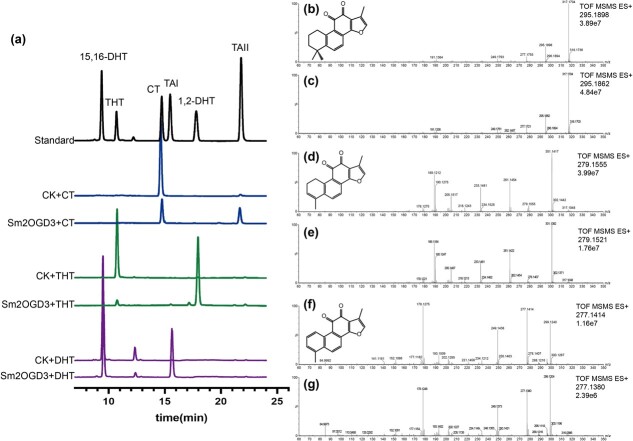
*In vitro* enzyme activity assay of Sm2OGD3 recombinant proteins. **a** UPLC analysis of the reaction products catalyzed by Sm2OGD3 using CT, THT, or 15,16-DHT as the substrate, respectively. The crude extracts from *Escherichia coli* cells carrying empty pET-30a vector were used as the control (CK). Cofactors were 160 μM 2-oxoglutarate and 50 μM ferrous sulfate. **b**–**g** Comparison of the MS–MS data between reaction products and standards. The MS–MS spectra of standards TAII (**b**), 1,2-DHT (**d**), TAI (**f**), the reaction products of Sm2OGD3 + CT with retention time 21.69 min (**c**), Sm2OGD3 + THT with retention time 17.78 min (**e**) and Sm2OGD3 + DHT with retention time 15.46 min (**g**) are shown. Response value and molecular weight of quasimolecular ion are shown at the top-right corner. The chemical structures of TAII, 1,2-DHT and TAI are also shown. CT, cryptotanshinone; TAII, tanshinone IIA; 15,16-DHT, 15,16-dihydrotanshinone I; TAI, tanshinone I; THT, 1,2,15,16-tetrahydrotanshinone I; 1,2-DHT, 1,2-dihydrotanshinone I.

To test whether Sm2OGD3 protein can catalyze 15,16-dehydrogenation of tanshinones, full-length *Sm2OGD3* cDNA was amplified from the roots of 99–3. It was then cloned into an *Escherichia coli* expression vector pET-30a (Fig. S8a, see online supplementary material). Recombinant protein of Sm2OGD3 was expressed in *E. coli* strain BL21(DE3) (Fig. S8b, see online supplementary material). Enzyme activity was analyzed *in vitro*. *E. coli* harboring the empty pET-30a vector was used as a control. The crude Sm2OGD3 enzymes were incubated with CT, THT, or 15,16-DHT that has a single bond at C-15,16. The ethyl acetate extracts obtained from these incubations were analysed using LC–MS. MS–MS data of samples were compared to standards. The results showed that CT, THT, and 15,16-DHT were converted into TAII, 1,2-DHT, and TAI, respectively (Fig. 5). No such conversion was found for the crude extract proteins of *E. coli* cells carrying the empty pET-30a vector (Fig. 5). It suggests that the Sm2OGD3 recombinant protein can convert the single bond at C-15,16 of tanshinones to double bond.

Furthermore, we examined the effect of different components of the reaction system on the enzyme activity using DHT as the substrate (Fig. S9, see online supplementary material). When L-ascorbate was absent, elevated levels of 2-oxoglutarate and Fe^2+^ did not significantly contribute to the reaction rate (reaction 1, 2, 6, and 7). The reaction rate for the removal of Fe^2+^ alone was similar to that for the removal of L-ascorbate alone (reaction 3). When both Fe^2+^ and L-ascorbate were present in the system, the reaction rate was significantly higher (reaction 4 and 5). To optimise the reaction conditions, protein activity was evaluated at different temperatures (20°C, 30°C, and 37°C) and pH values (5.8, 6.5, 7.3, and 8.0) using CT as the substrate. The results showed that the optimal conditions were 30°C and pH 8.0 (Fig. S10, see online supplementary material). The enzymatic rate toward each substrate was measured at different substrate concentrations using the optimal conditions and reaction system five. The *Km* values of Sm2OGD3 toward CT, 15,16-DHT, and THT were 32.15, 55.42, and 20.92 μM, respectively and the estimated *V*_max_ values were 0.3318, 1.228, and 0.7303 μM/min, respectively (Fig. S11, see online supplementary material). The results showed that 2OGD3 had a higher affinity for THT, followed by CT. In the roots of *S. miltiorrhiza*, the content of CT was higher than that of THT and the content of TAII was higher than that of 1,2-DHT (Fig. 1c). The content of the products was similar to that of the corresponding substrates, respectively (Fig. 1c). This implies that the conversion efficiency of Sm2OGD3 seems to be more dependent on the substrate concentration.

### Intact *Sm2OGD3* recovered the production of tanshinones with a double bond at C-15,16 in line shh transgenic hairy roots

**Figure 6:**
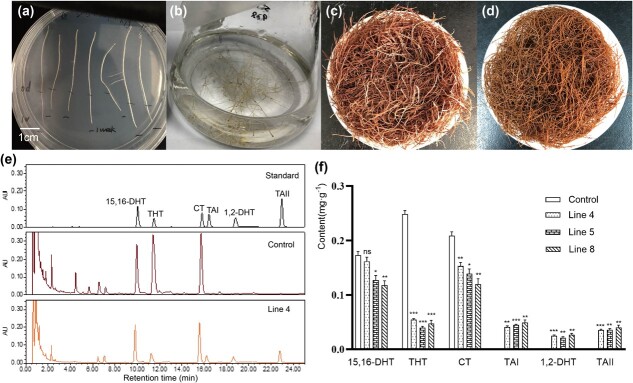
UPLC analysis of transgenic *Salvia miltiorrhiza* hairy roots. **a**–**d** Growth of transgenic hairy roots. **a** Transgenic hairy roots with hygromycin-resistant grow normally on screening medium with hygromycin 50 mg l^−1^ for one week. **b** Transgenic hairy roots grow on liquid medium. **c** Mature hairy roots of transgenic line 8. **d** Mature hairy roots of control (shh). **e** Comparative analysis of mature hairy roots from transgenic line 4 and control using UPLC. **f** Comparative analysis of cryptotanshinone (CT), tanshinone IIA (TAII), 15,16-dihydrotanshinone I (15,16-DHT), tanshinone I (TAI), 1,2,15,16-tetrahydrotanshinone I (THT) and 1,2-dihydrotanshinone I (1,2-DHT) contents in transgenic hairy roots and control. The error bars denote 1SE. ^*^P < 0.05; ^**^P < 0.01; ^***^P < 0.001; ns, not significant.

*Agrobacterium rhizogenes* strain ACCC10060 containing recombinant vector with intact *Sm2OGD3* ORF was used to transform *S. miltiorrhiza* line shh for the generation of transgenic hairy roots. ACCC10060 strain without *Sm2OGD3* ORF was used as a control. UPLC analysis showed that TAI, 1,2-DHT, and TAII were undetected in shh hairy roots without the intact *Sm2OGD3*. The results were consistent with those from the roots of shh plants (Fig. 1). In the transgenic hairy roots with intact *Sm2OGD3* (Fig. 6a–d), tanshinones with a double bond at C-15,16 were accumulated (Fig. 6e). The average contents of TAI, 1,2-DHT, and TAII in lines 4, 5, and 8 were 0.045, 0.024, and 0.037 mg.g^−1^ fresh weight, respectively. Furthermore, the contents of tanshinones with a single bond at C-15,16 were significantly reduced. CT, 15,16-DHT, and THT were decreased to 1.28-, 5.25-, and 1.52-folds of control, respectively (Fig. 6f).

### Orthologous genes of *Sm2OGD3* in *salvia* species

**Figure 7:**
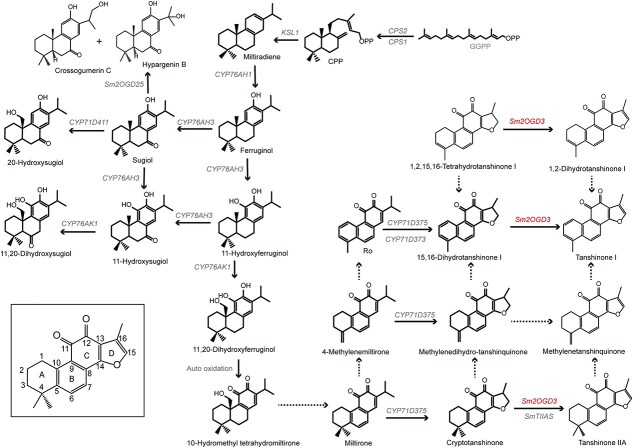
Proposed tanshinone biosynthesis pathway. Solid arrows indicate confirmed reactions. Dashed arrows indicate hypothetical reactions.

When we were conducting this study, a Sm2-ODD14, which was designated as *S. miltiorrhiza* tanshinone IIA synthase (SmTIIAS), was identified to convert CT and isocryptotanshinone to TAII and isotanshione IIA, respectively (Fig. 7) [18]. The ORF of *SmTIIAS* showed 98.6% identity with *Sm2OGD3*, and the amino acid sequence of SmTIIAS showed 97.3% identity with Sm2OGD3. Both Sm2OGD3 and SmTIIAS could convert CT to tanshinone TAII. However, other substrates that they could accept were different. SmTIIAS showed strict substrate specificity to CT and isocryptotanshinone. Differently, Sm2OGD3 showed a broader substrates spectrum. It could accept CT, 15,16-DHT, and THT as substrates. First, to clarify the identity of SmTIIAS and Sm2OGD3, we respectively identified these two genes in two *S. miltiorrhiza* lines (993 and DSS3) and several *salvia* plants (*S. bowleyana* [34], *S. splendens* [35] and *Salvia officinalis* [36]) with published genomes. Genomic location information of *Sm2OGD3* and *SmTIIAS *is identical in all five *salvia* plants (Table S13, see online supplementary material). This suggests that *Sm2OGD3* and *SmTIIAS* are not both present in *S. miltiorrhiza*. They are just alleles in different *S. miltiorrhiza* lines.

To further confirm the catalytic function of these alleles and homologs, we synthesized *SmTIIAS* and these newly identified genes (Table S12 and Fig. S12, see online supplementary material). Enzymatic reactions show that, as with Sm2OGD3, SmTIIAS, Sm2OGD3_DSS3, and Sb2OGD3 from *S. bowleyana* could all convert CT, 15,16-DHT, and THT to TAII, TAI, and 1,2-DHT, respectively (Fig. S13, see online supplementary material). Ss2OGD3_like from *S. splendens* and So2OGD3_like from *S. officinalis* failed to catalyze 15,16-dehydrogenation (Fig. S13, see online supplementary material). This result is consistent with the metabolite phenotype of the plants. The roots of *S. bowleyana* could be used as a substitute for *S. miltiorrhiza* due to its high contents of tanshinones [34]. The content of CT, TAII, and TAI are extremely low or undetected in *S. officinalis* and *S. splendens* [35, 36].

## Discussion

*S. miltiorrhiza* has attracted widespread research interests due to its high economic and therapeutic value and has developed as a model system for medicinal plant biology [1]. Numerous transcriptome and sRNAome data has been generated from various *S. miltiorrhiza* tissues with or without treatments [1]. The whole genomes of four *S. miltiorrhiza* lines with brick-red roots have been sequenced and assembled [15, 27–29]. In this study, a novel *S. miltiorrhiza* line, named shh, was sequenced and subsequently assembled through a combination of different technologies, such as Illumina paired-end, 10X Genomics, and PacBio SMART sequencing. It resulted in the acquirement of a chromosome level assembly of shh with high degree of continuity and completeness. The assembled shh genome has a total length of 530.97 Mb, 93.51% of which was anchored into eight pseudochromosomes with a super-scaffold N50 of 60.28 Mb and a maximum scaffold length of 77.73 Mb. It gives a strong groundwork for functional genomic analysis of bioactive compound biosynthesis in *S. miltiorrhiza*. Due to poor quality of the 99–3 genome assembly, we selected the *S. miltiorrhiza* line DSS3 genome assembled to the chromosome level for blastp-based collinearity analysis with the shh genome using MCScanX. The reasults from MCScanX showed that the encoding genes in shh assembly and DSS3 assembly had a high degree of genome-wide collinearity (Fig. S14, see online supplementary material). *Sm2OGD3_DSS3* (ID: GWHGAOSJ020467) is located in DSS3_7 pseudochromosomes and it does not have any alignments. *Sm2OGD3m* is located in SHH_7 pseudochromosomes (position: 311089–309 911). However, there did not appear to be some collinear blocks in the shh pseudochromosomes for some segments on the DSS3_3 and DSS3_5 pseudochromosomes (Fig. S14, see online supplementary material). To follow up with a more comprehensive analysis of genomic structural variation, we need to perform DNA sequence-based genome global alignment and obtain second and third generation sequencing reads of DSS3 and shh for identification by means of more systematic bioinformatic analysis.

Different from normal *S. miltiorrhiza* lines with brick-red roots, shh is characteristic with orange roots. Metabolomic and UPLC analyses showed increased contents of tanshinones with a single bond at C-15,16 and significantly decreased contents of those with a double bond at C-15,16. The variations of tanshinone compounds in shh were similar to that of the other *S. miltiorrhiza* line with orange roots [25]. Previously, systematic analysis of transcriptome profiles showed that four genes related to endoplasmic reticulum (ER)-associated degradation of proteins were up-regulated in orange roots [25] and no genes were down-regulated in the CYP, NADP/NAD-dependent dehydrogenases, and reductases [25]. Zhan *et al.* [25] proposed that, in orange *S. miltiorrhiza* roots, the decrease of tanshinones with a furan D-ring could be caused by incorrect folding and endoplasmic reticulum (ER)-associated degradation of enzymes catalyzing 15,16-dehydrogenation. In this study, the enzyme catalyzing 15,16-dehydrogenation has been shown to be Sm2OGD3. Moreover, Sm2OGD3 in shh lost the second exon and forms a truncated protein Sm2OGD3m. It seems to indicate that the accumulation of tanshinone compounds with a single bond at C-15,16 and the reduction of those with a double bond at C-15,16 could be caused by loss of Sm2OGD3 function in shh. According to Zhan *et al.* [25], we identify these genes except *c91931_g1* in *S. miltiorrhiza* 99–3 (Table S14, see online supplementary material). Among them, *SMil_00017121* was significantly up-regulated in shh with log2FoldChange 2.6 and padj 0.0015. *SMil_00006173* and *SMil_00005538* were not the differentially expressed genes between 99–3 and shh root periderm. Gene *c80749_g2* was annotated as ER luminal binding protein (BIP) [25]. Similarly, *SMil_00017121* was annotated as luminal-binding protein belonging to heat shock proteins (HSP70) against the NR and SMART databases. These results seem to imply that misfolding of the truncated protein in shh triggers endoplasmic reticulum-associated protein degradation. In our study, *Sm2OGD3* was mutated in the orange root of shh, so are the same mutations present in other orange roots of *S. miltiorrhiza* lines? Our findings are an important contribution to the analysis of the mechanism of *S. miltiorrhiza* orange roots formation.

The plant 2OGD superfamily is usually divided into three classes, including DOXA, DOXB, and DOXC [23]. Members of the DOXA and DOXB classes are involved in primary metabolic networks, such as DNA repair, histone demethylation, and post-translational modification [23]. Members of the DOXC class not only participate in the conserved pathways of phytohormone metabolism but also contribute to the production of highly specialized metabolites, such as phenolic acids and alkaloids [21]. *S. miltiorrhiza* line 99–3 has 132 Sm2OGDs, of which three belong to DOXA, eight belong to DOXB, 118 belong to DOXC, and the other three are unclassified [16]. The 118 DOXC members can be further divided into 13 clades, including ACO, AOP, CODM/NCS, D4H/GSLOH/BX6, DAO, F3H, FLS/ANS, GA2ox, GA3ox, GA20ox, H6H, S3H, and unknown [16]. *Sm2OGD3* was assigned to the D4H/GSLOH/BX6 clade. The 12 significantly down-regulated *Sm2OGD* gene models in shh belong to six clades of the DOXC classes. Through genomic and transcriptomic comparison, *in vitro* protein assay and *in vivo* complementation assay, we found that Sm2OGD3 was responsible for 15,16-dehydrogenation of tanshinones. In addition, several homologous proteins of Sm2OGD3 from other *S. miltiorrhiza* lines and *S. bowleyana* also could convert CT, 15,16-DHT, and THT into TAII, TAI, and 1,2-DHT, respectively. The results confirmed the catalytic roles of Sm2OGD3 in 15,16-dehydrogenation, a key reaction in the tanshinone biosynthetic pathway (Fig. 7). Identification of the enzyme catalyzing this reaction is significant for elucidation of tanshinone biosynthesis and regulatory mechanisms. The results can also contribute to synthetic biology of tanshinones, a class of medicinally and economically important bioactive compounds.

## Materials and methods

### Library construction and sequencing

Genomic DNA (gDNA) of shh leaves was isolated by the cetyl trimethyl ammonium bromide (CTAB) method as per the manufacturer’s protocol [37]. For Illumina sequencing, a short-insert library was constructed by randomly broking gDNA into fragments using Covaris ultrasonic fragmentation, followed by end repair, addition of poly-A, ligation of adapters, template purification, and PCR amplification. The constructed library was sequenced using Illumina paired-end sequencing technology (San Diego, CA, USA). For PacBio SMRT sequencing, a SMRTbell library was first built by ligating hairpin adapters to the gDNA, and then primers and polymerase were added. DNA sequencing was running on the PacBio Sequel instrument (Menlo Park, CA, USA). For 10× Genomics libraries, Illumina P5 adapters, barcodes, Illumina sequencing primers, and random primers linked to Gel Beads and then Gel Beads combined with DNA fragments and wrapped in oil. After PCR amplification, Illumina P7 adapters were added to the libraries for illumina sequencing.

### Genome assembly and assessment

Based on PacBio reads, pre-assembled contigs were generated using FALCON (https://github.com/PacificBiosciences/FALCON/). Phasing of the resulting contigs was carried out using FALCON-Unzip. The contigs were polished with PacBio reads using the Quiver software [38]. Illumina reads were analysed using pilon [39] to improve the quality of assembly. Because of the high heterozygosity of *S. miltiorrhiza* genome, the genome assembly was de-hybridized using the purge_haplotigs [40]. The filtered assembly was scaffolded on the basis of 10$\times$ Genomics Linked-reads using fragScaff software [41]. To assess the integrity of the assembly and the homogeneity of the sequencing, Illumina reads were mapped onto the assembly via BWA software [31] and calculated for mapping rate, genome coverage, and sequencing depth. CEGMA [33] and BUSCO [32] were applied to assess the completeness of the assembly.

### Hi-C library construction and assignment of the chromosome

To construct Hi-C library, the gDNA was fixed with paraformaldehyde, digested and biotin-labeled. Cells were treated with paraformaldehyde to immobilise the DNA. The cross-linked DNA was then cut using restriction endonucleases. For end repair, biotin was added to label the oligonucleotide ends. Ligation of adjacent chromatin DNA fragments with T4 DNA ligase. Proteinase was added to digest the ligase and uncross-link the proteins and DNA. Genomic DNA was extracted and randomly broken into fragments of 350 bp. DNA marked with biotin was captured by affinity beads, followed by end-repair, A-tailing, adapter ligation, PCR amplification, and template purification. The libraries were sequenced on Illumina HiSeq PE150.

The filtered reads were mapped to the draft assembly. Duplicated mapping and unmapping reads were cut out using SAMtools [42] with the parameter rmdup. The reads attached to the ligation sites were picked out for ancillary assembly. The contigs were assigned, ordered, and oriented using LACHESIS (version-201 701) (http://shendurelab.github.io/LACHESIS/).

### Genome annotation

Repeat elements were annotated based on homology alignment and *de novo* search [43]. Homolog prediction was carried out through alignment of the Repbase database with the genome assembly using RepeatMasker (http://www.repeatmasker.org/). Tandem Repeat was extracted from the genome assembly using TRF (http://tandem.bu.edu/trf/trf.html). *De novo* repetitive element database was constructed through *ab initio* prediction using LTR_FINDER [44], RepeatScou, and RepeatModeler. All of the repeat sequences with lengths greater than 100 bp and gap ‘N’ less than 5% were used to construct the raw transposable element (TE) library. Repbase and TE library were handled by UCLUST [45] to yield a non-redundant library which was given to RepeatMasker for repeat identification.

Homology-based, *ab initio* and RNA-Seq assisted predictions were used to annotate gene models. For homology-based prediction, protein sequences from *Arabidopsis thaliana*, *S. lycopersicum*, *P. ginseng*, *S. indicum*, *S. splendens*, and *S. miltiorrhiza* line 99–3 were downloaded and aligned with shh genome by TblastN [46] (v2.2.26; *E*-value ≤1e−5). Matched proteins were mapped to the homologous genomes for gene splicing prediction using GeneWise [47] (v2.4.1). Augustus (v3.2.3), Geneid [48], Genescan [49], GlimmerHMM [50], and SNAP [51] were used in *ab initio* gene prediction pipeline. To optimize the gene structure annotation, RNA-seq reads from different organs were aligned with shh genome assembly using TopHat [52]. The results from alignment were used as input for genome-based transcript assembly using Cufflinks [53]. Non-redundant reference gene collection was generated through merging gene models predicted by three methods using EvidenceModeler [54]. The PASA was used to improve the gene structures [55].

The functions of predicted protein sequences were analysed against the Swiss-Prot, NR, and KEGG databases using Blastp with a threshold of *E*-value ≤1e-5. Motifs and domains were assigned by InterProScan70 (v5.31) [56] through searching the predicted proteins against Pfam and InterPro databases. Gene Ontology (GO) IDs of each gene were accredited based on the related InterPro entry.

### Comparative genomic analysis

The genome and annotation data for *S. miltiorrhiza*_99–3, *S. miltiorrhiza*_unknown, *S. splendens*, *E. guttata*, *S. indicum*, *U. gibba*, *Olea europaea*, *S. lycopersicum*, *S. tuberosum*, *P. ginseng*, *D. carota*, *A. thaliana*, and *P. trichocarpa* were downloaded. The relationship of orthologous genes in shh and the other 13 plant species was inferred through running all-against-all blastp program. Gene family clustering was performed using OrthoMCL [57] with ‘-mode 3 -inflation 1.5’. The software CAFE was applied to measure the expansion and contraction of orthologous gene families [58]. For each single-copy gene family, an alignment was produced using Muscle [59]. Ambiguously aligned posiions were trimmed by Gblocks [60]. Phylogenomic tree was constructed with RAxML (v.8.2.12). Divergence times were estimated based on the mcmctree program implemented in PAML [61] with default parameters. Syntenic blocks identified within *S. miltiorrhiza*_shh by MCScanX [62] were used for WGD analysis. The codeml program in the PAML package was used to estimate *K*s for all pairwise paralogous genes.

### Metabolite analysis

Fresh powder (0.1 g) was weighed into 1 ml of methanol, followed by sonicate for 20 min, and centrifuged at 8000 rpm for 5 min. The extraction was repeated once. The two supernatants from extracts were combined and filtered using a 0.22 μm microporous membrane. Metabolites were quantified using ultra-high-performance liquid chromatography (UPLC, Waters, MA, USA) equipped with a C18 column (1.7 μm, 2.1 × 100 mm; Waters). The detection wavelength, column temperature and flow rate were maintained at 270 nm, 25°C, and 0.3 ml min^−1^, respectively. The mobile phase was comprised of water with 0.1% (v/v) formic acid (A) and 100% acetonitrile (B). The gradient elution was performed as follows: 0 min–5 min, 40% B; 5 min–25 min, 40%–60% B.

For metabolomic analysis, Dionex Ultimate 3000 UPLC coupled with a Thermo Syncronis C18 column (2.1 mm × 100 mm, 1.7 μm) was used. A quality control sample mixing all samples is used to monitor the stability of the system. Q Exactive Orbitrap mass spectrometry equipped with electrospray ionization (ESI) source was performed to analyse metabolite profile. The settings were as follows: simultaneous positive and negative ion scanning mode; ion spray voltage (IS), 2.8 kV; Sheath gas flow rate, 35 arb; flow rate of the auxiliary gas, 10 arb; and capillary temperature, 320°C. A full scan with a resolution of 70 000 and a scanning range of 70–1050 (m/z) was adopted. The resolution of secondary data dependence scan (full MS/dd-MS) was 17 500. The Stepped NCE values are 20 V, 40 V, and 60 V.

### Analysis of genes involved in tanshinone biosynthesis

To identify the genes participating in the MVA and MEP pathways, the related protein sequences from *A. thaliana* and *S. miltiorrhiza* were used as queries to search against the shh genome assembly using BLASTP-algorithm with an *E*-value ≤1e-5. The gene sequences encoding *S. miltiorrhiza* CPS, KSL, CYP76AH1, CYP76AH3, CYP76AK1, CYP71D411, CYP71D375, and CYP71D373 were downloaded from NCBI. The *2OGD* gene family sequences were acquired from the genome annotation file of *S. miltiorrhiza_*99–3*.* These gene sequences were used as baits to identify the corresponding genes in shh genome with the filtering parameters, *E*-value ≤1e-50, identity ≥80%, and coverage ≥80%. The candidates were submitted to pfam and SMART databases for domain and motif analysis. Proteins with same conserved domains were considered to be homologs. Transcriptome data published previously [22] were analysed for the expression level of tanshinone biosynthesis-related genes using align-free software Salmon [63]. The heat map was generated using TBtools [64].

### *In vitro* analysis of Sm2OGD3 protein activity

The open reading frame (ORF) of *Sm2OGD3* from mature roots of 99–3 were PCR-amplified and cloned into the PET-30a vector. *E. coli* strain BL21(DE3) harboring the recombinant plasmid was grown in LB medium at 37°C until OD_600_ reached to 0.6. Subsequently, the culture temperature was cooled to 20°C. Induction of recombinant proteins was carried out using 0.5 mM isopropyl β-D-thiogalactopyranoside (IPTG) at 20°C for 20 h. Cells from 100 ml of culture were harvested by centrifugation and resuspended at 4°C in 15 ml PBS buffer (pH 7.2–7.4) containing Na_2_HPO_4_, NaH_2_PO_4_, and NaCl [65]. Cells were lysed by sonication for 30 min (lysed for 3 s, paused for 10 s) with 187.5 watt. The crude extracts were obtained by centrifugation and then incubated with 160 μM 2-oxoglutarate, 50 μM ferrous sulfate, and 10 to 100 μM substrate in a final volume of 0.5 ml PBS buffer for 0.5–1 h at 30°C [65]. As a negative control, proteins were similarly prepared from cells harboring an empty PET-30a plasmid. Reaction products were extracted twice in 0.5 ml ethyl acetate and then evaporated using a nitrogen blower. The residue was dissolved in 0.2 ml of methanol, filtered and analysed using LC–MS (UPLC-electrospray ionization (ESI)–MS, Waters). MS–MS data were analysed using MssLynx V4.1 software (Waters).

### Transgenic analysis of *Sm2OGD3*

*Sm2OGD3* ORF were PCR-amplified and cloned into pTOPO-Blunt vector. After verification using sanger sequencing, the ORF was digested with *Bst*E II and *Nco* I and then inserted into pCAMBIA1391 vector. *A. rhizogenes* strain ACCC10060 was utilized for plant transformation. Transgenic hairy roots were generated according the Wei’s procedure [66]. The forward primer 35S-F and the reverse primer Sm2OGD3-R were used for PCR identification of *Sm2OGD3* transgenic lines. PCR-positive hairy roots were transferred to 6,7-V liquid medium and sub-cultured every 3 weeks. After three months, hairy roots were harvested and frozen in liquid nitrogen. The frozen samples were ground into 0.2 g powders and extracted with 1 ml methanol. The extracts were evaporated and re-dissolved in 0.2 ml methanol. The filtrates were analyzed using UPLC.

## Acknowledgements

We are thankful for financial support from the CAMS Innovation Fund for Medical Sciences (CIFMS) (2021-I2M-1-029) and the National Natural Science Foundation of China (81773836).

## Author contributions

S.L. conceived and designed the study. X.P. and Y.C. conducted experiments and acquired data. X.P. and S.L. analysed and interpreted data. C.L., X.Q., X.C., F.M., and S.Z. helped with experiments and data analysis. X.L. provided plant materials. X.P. and S.L. wrote and revised the manuscript. All authors approved the final version of the manuscript.

## Data availability

The data that supports the findings of this study are available in the supplementary material of this article. The genome sequence of *S. miltiorrhiza* line shh is available at NCBI BioProject PRJNA903271. The annotation information has been uploaded to the National Genomics Data Center (https://ngdc.cncb.ac.cn/) under the accession number WGS038566.

## Conflict of interest statement:

The authors declare no conflicts of interest.

## Supplementary data

Supplementary data is available at *Horticulture Research* online.

## Supplementary Material

Web_Material_uhad069

## References

[1] Lu S . Biosynthesis and regulatory mechanisms of bioactive compounds in *Salvia miltiorrhiza*, a model system for medicinal plant biology. Crit Rev Plant Sci. 2021;40:243–83.

[2] Su CY , Ming QL, Rahman K et al. *Salvia miltiorrhiza*: traditional medicinal uses, chemistry, and pharmacology. Chin J Nat Med. 2015;13:163–82. 25835361 10.1016/S1875-5364(15)30002-9

[3] Jiang Z , Gao W, Huang L. Tanshinones, critical pharmacological components in. Front Pharmacol. 2019;10:202. 30923500 10.3389/fphar.2019.00202 PMC6426754

[4] Xu Z , Peters RJ, Weirather J et al. Full-length transcriptome sequences and splice variants obtained by a combination of sequencing platforms applied to different root tissues of *Salvia miltiorrhiza* and tanshinone biosynthesis. Plant J. 2015;82:951–61. 25912611 10.1111/tpj.12865

[5] Li S , Zhu N, Tang C et al. Differential distribution of characteristic constituents in root, stem and leaf tissues of *Salvia miltiorrhiza* using MALDI mass spectrometry imaging. Fitoterapia. 2020;146:104679. 32619463 10.1016/j.fitote.2020.104679

[6] Wu YB , Ni ZY, Shi QW et al. Constituents from *salvia* species and their biological activities. Chem Rev. 2012;112:5967–6026. 22967178 10.1021/cr200058f

[7] Meim XD , Cao YF, Che YY et al. Danshen: a phytochemical and pharmacological overview. Chin J Nat Med. 2019;17:59–80. 30704625 10.1016/S1875-5364(19)30010-X

[8] Gao W , Hillwig ML, Huang L et al. A functional genomics approach to tanshinone biosynthesis provides stereochemical insights. Org Lett. 2009;11:5170–3. 19905026 10.1021/ol902051v PMC2776380

[9] Ma Y , Yuan L, Wu B et al. Genome-wide identification and characterization of novel genes involved in terpenoid biosynthesis in *Salvia miltiorrhiza*. J Exp Bot. 2012;63:2809–23. 22291132 10.1093/jxb/err466 PMC3346237

[10] Cheng Q , Su P, Hu Y et al. RNA interference-mediated repression of SmCPS (copalyldiphosphate synthase) expression in hairy roots of *Salvia miltiorrhiza* causes a decrease of tanshinones and sheds light on the functional role of SmCPS. Biotechnol Lett. 2014;36:363–9. 24078134 10.1007/s10529-013-1358-4

[11] Shi M , Luo X, Ju G et al. Increased accumulation of the cardio-cerebrovascular disease treatment drug tanshinone in *Salvia miltiorrhiza* hairy roots by the enzymes 3-hydroxy-3-methylglutaryl CoA reductase and 1-deoxy-D-xylulose 5-phosphate reductoisomerase. Funct Integr Genomics. 2014;14:603–15. 24913677 10.1007/s10142-014-0385-0

[12] Cui G , Duan L, Jin B et al. Functional divergence of diterpene syntheses in the medicinal plant *Salvia miltiorrhiza*. Plant Physiol. 2015;169:1607–18. 26077765 10.1104/pp.15.00695 PMC4634056

[13] Guo J , Zhou YJ, Hillwig ML et al. CYP76AH1 catalyzes turnover of miltiradiene in tanshinones biosynthesis and enables heterologous production of ferruginol in yeasts. Proc Natl Acad Sci U S A. 2013;110:12108–13. 23812755 10.1073/pnas.1218061110 PMC3718111

[14] Guo J , Ma X, Cai Y et al. Cytochrome P450 promiscuity leads to a bifurcating biosynthetic pathway for tanshinones. New Phytol. 2016;210:525–34. 26682704 10.1111/nph.13790 PMC4930649

[15] Ma Y , Cui G, Chen T et al. Expansion within the CYP71D subfamily drives the heterocyclization of tanshinones synthesis in *Salvia miltiorrhiza*. Nat Commun. 2021;12:685. 33514704 10.1038/s41467-021-20959-1 PMC7846762

[16] Xu Z , Song J. The 2-oxoglutarate-dependent dioxygenase superfamily participates in tanshinone production in *Salvia miltiorrhiza*. J Exp Bot. 2017;68:2299–308. 28398557 10.1093/jxb/erx113 PMC5447875

[17] Hu Z , Ren L, Bu J et al. Functional characterization of a 2OGD involved in Abietane-type diterpenoids biosynthetic pathway in *Salvia miltiorrhiza*. Front Plant Sci. 2022;13:947674. 35873989 10.3389/fpls.2022.947674 PMC9301305

[18] Song JJ , Fang X, Chen YL et al. A 2-oxoglutarate-dependent dioxygenase converts dihydrofuran to furan in *salvia* diterpenoids. Plant Physiol. 2022;188:1496–506. 34893909 10.1093/plphys/kiab567 PMC8896610

[19] Islam MS , Leissing TM, Chowdhury R et al. 2-oxoglutarate-dependent oxygenases. Annu Rev Biochem. 2018;87:585–620. 29494239 10.1146/annurev-biochem-061516-044724

[20] Hagel JM , Facchini PJ. Expanding the roles for 2-oxoglutarate-dependent oxygenases in plant metabolism. Nat Prod Rep. 2018;35:721–34. 29488530 10.1039/c7np00060j

[21] Farrow SC , Facchini PJ. Functional diversity of 2-oxoglutarate/Fe(II)-dependent dioxygenases in plant metabolism. Front Plant Sci. 2014;5:524. 25346740 10.3389/fpls.2014.00524 PMC4191161

[22] Cheng AX , Han XJ, Wu YF et al. The function and catalysis of 2-oxoglutarate-dependent oxygenases involved in plant flavonoid biosynthesis. Int J Mol Sci. 2014;15:1080–95. 24434621 10.3390/ijms15011080 PMC3907857

[23] Kawai Y , Ono E, Mizutani M. Evolution and diversity of the 2-oxoglutarate-dependent dioxygenase superfamily in plants. Plant J. 2014;78:328–43. 24547750 10.1111/tpj.12479

[24] Chang Y , Wang M, Li J et al. Transcriptomic analysis reveals potential genes involved in tanshinone biosynthesis in *Salvia miltiorrhiza*. Sci Rep. 2019;9:14929. 31624328 10.1038/s41598-019-51535-9 PMC6797793

[25] Zhan Z , Fang W, Ma X et al. Metabolome and transcriptome analyses reveal quality change in the orange-rooted (Danshen) from cultivated field. Chin Med. 2019;14:42. 31592267 10.1186/s13020-019-0265-6 PMC6775661

[26] Su Y , Zhang J, Xu Z et al. Integrative analysis of metabolome and transcriptome reveals the mechanism of color formation in white root (*Salvia miltiorrhiza*). Ind Crop Prod. 2021;170:113784.

[27] Zhang G , Tian Y, Zhang J et al. Hybrid *de novo* genome assembly of the Chinese herbal plant danshen (*Salvia miltiorrhiza* Bunge). GigaScience. 2015;4:62. 26673920 10.1186/s13742-015-0104-3 PMC4678694

[28] Xu H , Song J, Luo H et al. Analysis of the genome sequence of the medicinal plant *Salvia miltiorrhiza*. Mol Plant. 2016;9:949–52. 27018390 10.1016/j.molp.2016.03.010 PMC5517341

[29] Song Z , Lin C, Xing P et al. A high-quality reference genome sequence of *Salvia miltiorrhiza* provides insights into tanshinone synthesis in its red rhizomes. Plant Genome. 2020;13:e20041. 33217202 10.1002/tpg2.20041 PMC12807052

[30] Burton JN , Adey A, Patwardhan RP et al. Chromosome-scale scaffolding of *de novo* genome assemblies based on chromatin interactions. Nat Biotechnol. 2013;31:1119–25. 24185095 10.1038/nbt.2727 PMC4117202

[31] Li H , Durbin R. Fast and accurate long-read alignment with burrows-wheeler transform. Bioinformatics. 2010;26:589–95. 20080505 10.1093/bioinformatics/btp698 PMC2828108

[32] Simão FA , Waterhouse RM, Ioannidis P et al. BUSCO: assessing genome assembly and annotation completeness with single-copy orthologs. Bioinformatics. 2015;31:3210–2. 26059717 10.1093/bioinformatics/btv351

[33] Parra G , Bradnam K, Korf I. CEGMA: a pipeline to accurately annotate core genes in eukaryotic genomes. Bioinformatics. 2007;23:1061–7. 17332020 10.1093/bioinformatics/btm071

[34] Zheng X , Chen D, Chen B et al. Insights into salvianolic acid B biosynthesis from chromosome-scale assembly of the *Salvia bowleyana* genome. J Integr Plant Biol. 2021;63:1309–23. 33634943 10.1111/jipb.13085

[35] Dong A , Xin H, Li Z et al. High-quality assembly of the reference genome for scarlet sage, *Salvia splendens*, an economically important ornamental plant. GigaScience. 2018;7:1–10. 10.1093/gigascience/giy068 PMC6030905 29931210

[36] Li C , Lei Y, Liu Y et al. The sage genome provides insight into the evolutionary dynamics of diterpene biosynthesis gene cluster in plants. Cell Rep. 2022;40:111236. 35977487 10.1016/j.celrep.2022.111236

[37] Porebski S , Bailey LG, BR B. Modification of a CTAB DNA extraction protocol for plants containing high polysaccharide and polyphenol components. Plant Mol Biol Report. 1997;15:8–15.

[38] Chin CS , Alexander DH, Marks P et al. Nonhybrid, finished microbial genome assemblies from long-read SMRT sequencing data. Nat Methods. 2013;10:563–9. 23644548 10.1038/nmeth.2474

[39] Walker BJ , Abeel T, Shea T et al. Pilon: an integrated tool for comprehensive microbial variant detection and genome assembly improvement. PLoS One. 2014;9:e112963. 25409509 10.1371/journal.pone.0112963 PMC4237348

[40] Roach MJ , Schmidt SA, Borneman AR. Purge Haplotigs: allelic contig reassignment for third-gen diploid genome assemblies. BMC Bioinformatics. 2018;19:460. 30497373 10.1186/s12859-018-2485-7 PMC6267036

[41] Adey A , Kitzman J, Burton JN et al. *In vitro*, long-range sequence information for *de novo* genome assembly via transposase contiguity. Genome Res. 2014;24:2041–9. 25327137 10.1101/gr.178319.114 PMC4248320

[42] Li H , Handsaker B, Wysoker A et al. The sequence alignment/map format and SAMtools. Bioinformatics. 2009;25:2078–9. 19505943 10.1093/bioinformatics/btp352 PMC2723002

[43] Price AL , Jones NC, Pevzner PA. *De novo* identification of repeat families in large genomes. Bioinformatics. 2005;21:i351–8. 15961478 10.1093/bioinformatics/bti1018

[44] Xu Z , Wang H. LTR_FINDER: an efficient tool for the prediction of full-length LTR retrotransposons. Nucleic Acids Res. 2007;35:W265–8. 17485477 10.1093/nar/gkm286 PMC1933203

[45] Edgar RC . Search and clustering orders of magnitude faster than BLAST. Bioinformatics. 2010;26:2460–1. 20709691 10.1093/bioinformatics/btq461

[46] Altschul SF , Madden TL, Schäffer AA et al. Gapped BLAST and PSI-BLAST: a new generation of protein database search programs. Nucleic Acids Res. 1997;25:3389–402. 9254694 10.1093/nar/25.17.3389 PMC146917

[47] Birney E , Clamp M, Durbin R. GeneWise and Genomewise. Genome Res. 2004;14:988–95. 15123596 10.1101/gr.1865504 PMC479130

[48] Parra G , Blanco E, Guigó R. GeneID in drosophila. Genome Res. 2000;10:511–5. 10779490 10.1101/gr.10.4.511 PMC310871

[49] Li R , Zhu H, Ruan J et al. *De novo* assembly of human genomes with massively parallel short read sequencing. Genome Res. 2010;20:265–72. 20019144 10.1101/gr.097261.109 PMC2813482

[50] Majoros WH , Pertea M, Salzberg SL. TigrScan and GlimmerHMM: two open source ab initio eukaryotic gene-finders. Bioinformatics. 2004;20:2878–9. 15145805 10.1093/bioinformatics/bth315

[51] Korf I . Gene finding in novel genomes. BMC Bioinformatics. 2004;5:59. 15144565 10.1186/1471-2105-5-59 PMC421630

[52] Trapnell C , Pachter L, Salzberg SL. TopHat: discovering splice junctions with RNA-Seq. Bioinformatics. 2009;25:1105–11. 19289445 10.1093/bioinformatics/btp120 PMC2672628

[53] Trapnell C , Williams B, Pertea G et al. Transcript assembly and quantification by RNA-Seq reveals unannotated transcripts and isoform switching during cell differentiation. Nat Biotechnol. 2010;28:511–5. 20436464 10.1038/nbt.1621 PMC3146043

[54] Haas BJ , Salzberg SL, Zhu W et al. Automated eukaryotic gene structure annotation using EVidenceModeler and the program to assemble spliced alignments. Genome Biol. 2008;9:R7. 18190707 10.1186/gb-2008-9-1-r7 PMC2395244

[55] Haas BJ , Delcher A, Mount SM et al. Improving the Arabidopsis genome annotation using maximal transcript alignment assemblies. Nucleic Acids Res. 2003;31:5654–66. 14500829 10.1093/nar/gkg770 PMC206470

[56] Zdobnov EM , Apweiler R. InterProScan--an integration platform for the signature-recognition methods in InterPro. Bioinformatics. 2001;17:847–8. 11590104 10.1093/bioinformatics/17.9.847

[57] Li L , Stoeckert CJ, Roos DS. OrthoMCL: identification of ortholog groups for eukaryotic genomes. Genome Res. 2003;13:2178–89. 12952885 10.1101/gr.1224503 PMC403725

[58] De Bie T , Cristianini N, Demuth JP et al. CAFE: a computational tool for the study of gene family evolution. Bioinformatics. 2006;22:1269–71. 16543274 10.1093/bioinformatics/btl097

[59] Edgar RC . MUSCLE: multiple sequence alignment with high accuracy and high throughput. Nucleic Acids Res. 2004;32:1792–7. 15034147 10.1093/nar/gkh340 PMC390337

[60] Talavera G , Castresana J. Improvement of phylogenies after removing divergent and ambiguously aligned blocks from protein sequence alignments. Syst Biol. 2007;56:564–77. 17654362 10.1080/10635150701472164

[61] Yang Z . PAML 4: phylogenetic analysis by maximum likelihood. Mol Biol Evol. 2007;24:1586–91. 17483113 10.1093/molbev/msm088

[62] Wang Y , Tang H, Debarry JD et al. MCScanX: a toolkit for detection and evolutionary analysis of gene synteny and collinearity. Nucleic Acids Res. 2012;40:e49. 22217600 10.1093/nar/gkr1293 PMC3326336

[63] Sahraeian SME , Mohiyuddin M, Sebra R et al. Gaining comprehensive biological insight into the transcriptome by performing a broad-spectrum RNA-seq analysis. Nat Commun. 2017;8:59. 28680106 10.1038/s41467-017-00050-4 PMC5498581

[64] Chen C , Chen H, Zhang Y et al. TBtools: an integrative toolkit developed for interactive analyses of big biological data. Mol Plant. 2020;13:1194–202. 32585190 10.1016/j.molp.2020.06.009

[65] Wei S , Zhang W, Fu R et al. Genome-wide characterization of 2-oxoglutarate and Fe(II)-dependent dioxygenase family genes in tomato during growth cycle and their roles in metabolism. BMC Genomics. 2021;22:126. 33602133 10.1186/s12864-021-07434-3 PMC7891033

[66] Wei T , Gao Y, Deng K et al. Enhancement of tanshinone production in hairy root cultures by metabolic engineering. Plant Methods. 2019;15:53. 31143241 10.1186/s13007-019-0439-3 PMC6532201

