# Supplementary material for: Chromosome-level genome assembly of Salvia miltiorrhiza with orange roots uncovers the role of Sm2OGD3 in catalyzing 15,16-dehydrogenation of tanshinones
Source: Hortic Res. 2023 Apr 13;10(6):uhad069. doi: 10.1093/hr/uhad069 (PMC10244880; doi:10.1093/hr/uhad069)
Supplement: Web_Material_uhad069 [file web_material_uhad069.zip › Supplementary Information (FigS1-S14).docx]

**
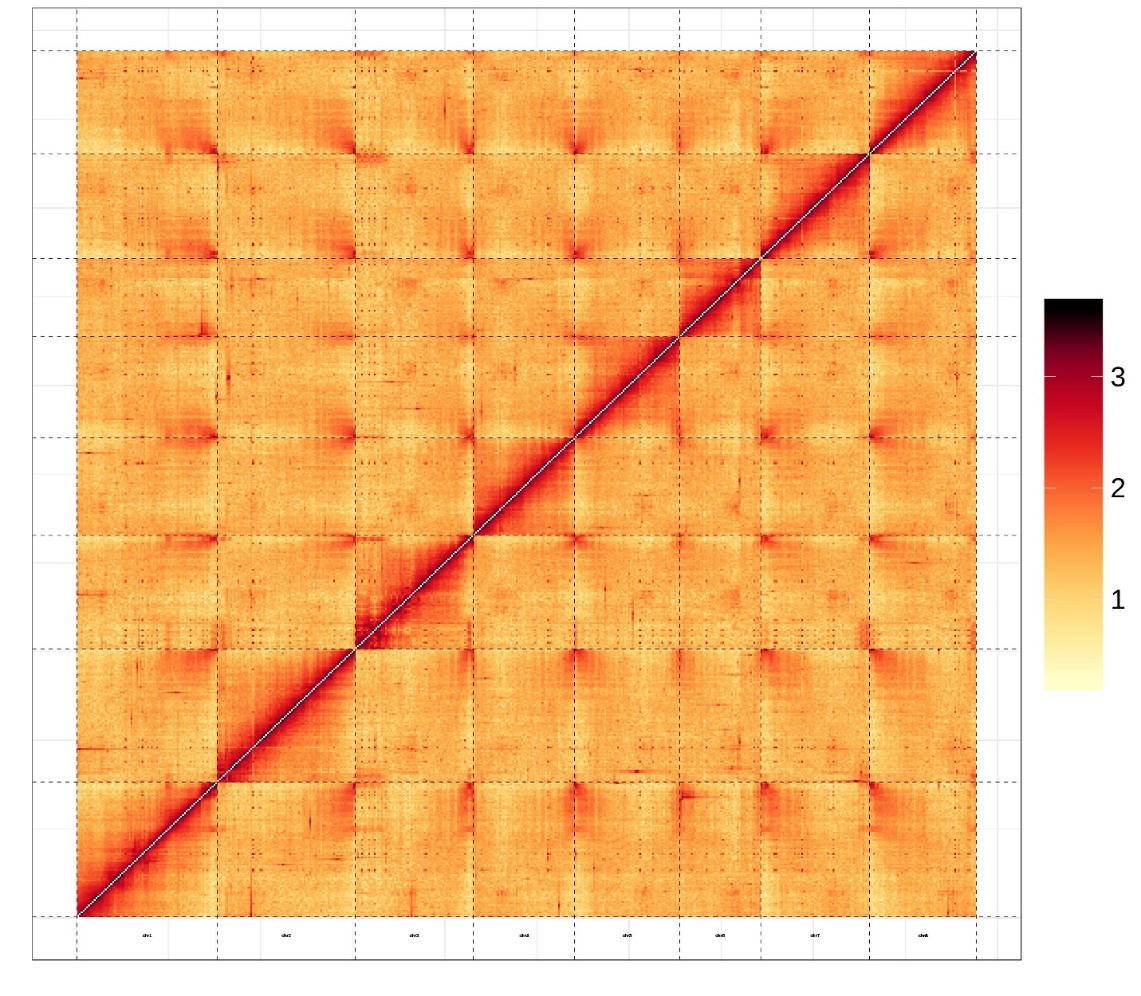
**

**Fig. S1** **Hi-C heatmap of shh assembly showing genome-wide all-by-all interactions**

**
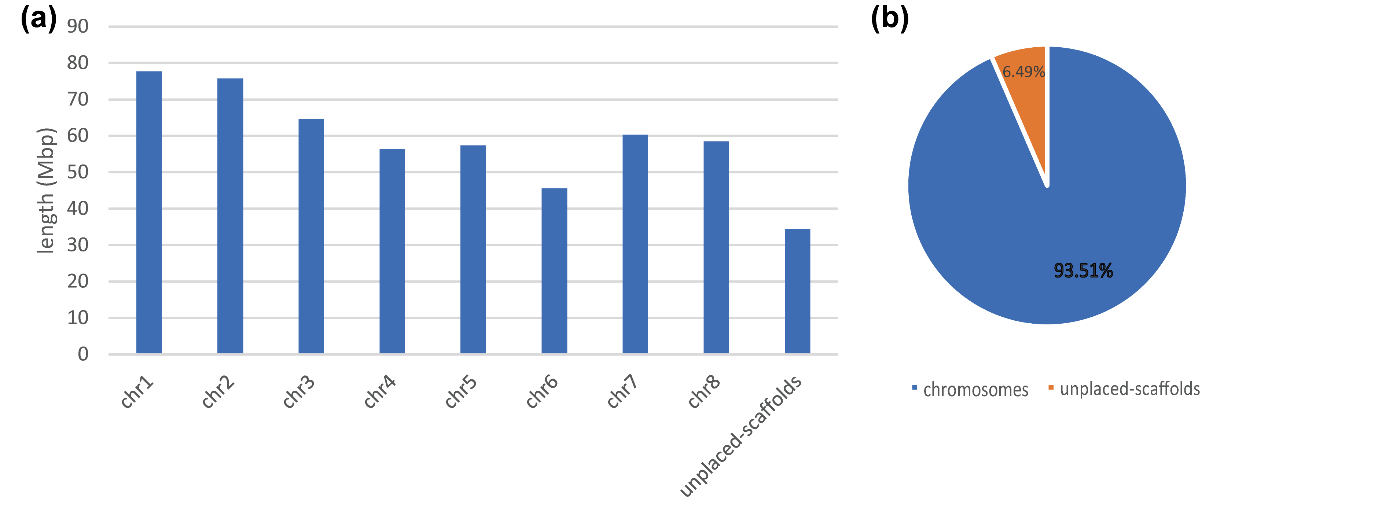
**

**Fig. S2** **Summary of the assembled shh genome.** (a) The size (Mbp) of 8 assembled chromosomes and unplaced scaffolds; (b) The proportions of 8 chromosomes and unplaced scaffolds.


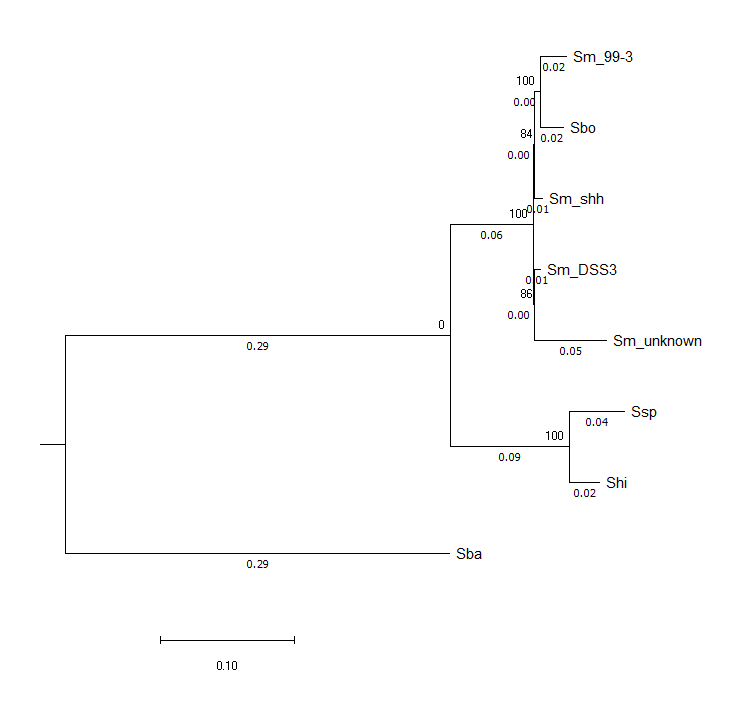


**Fig. S3** **Phylogenomic tree.** Sm, *Salvia miltiorrhiza*; Sbo, *Salvia bowleyana*; Ssp, *Salvia* *splendens*;Shi, *Salvia hispanica*; Sba, *Scutellaria baicalensis*. The value on the branch represents the number of Bootstrap supports; the branch length represents the rate of evolution.

**
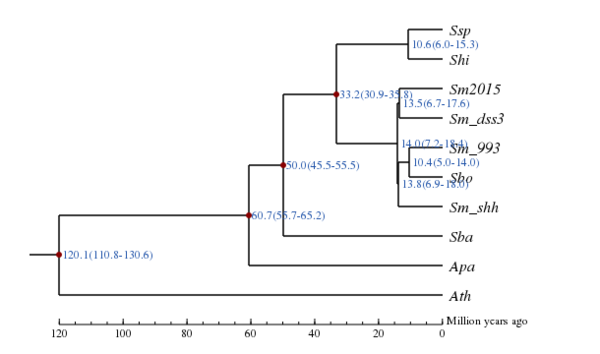
**

**Fig. S4** **Estimation of divergence time**. The number at the node position represents the divergence time of the species or ancestor of the species in millions of years, and the number in parentheses indicates the confidence range for that divergence time.

**
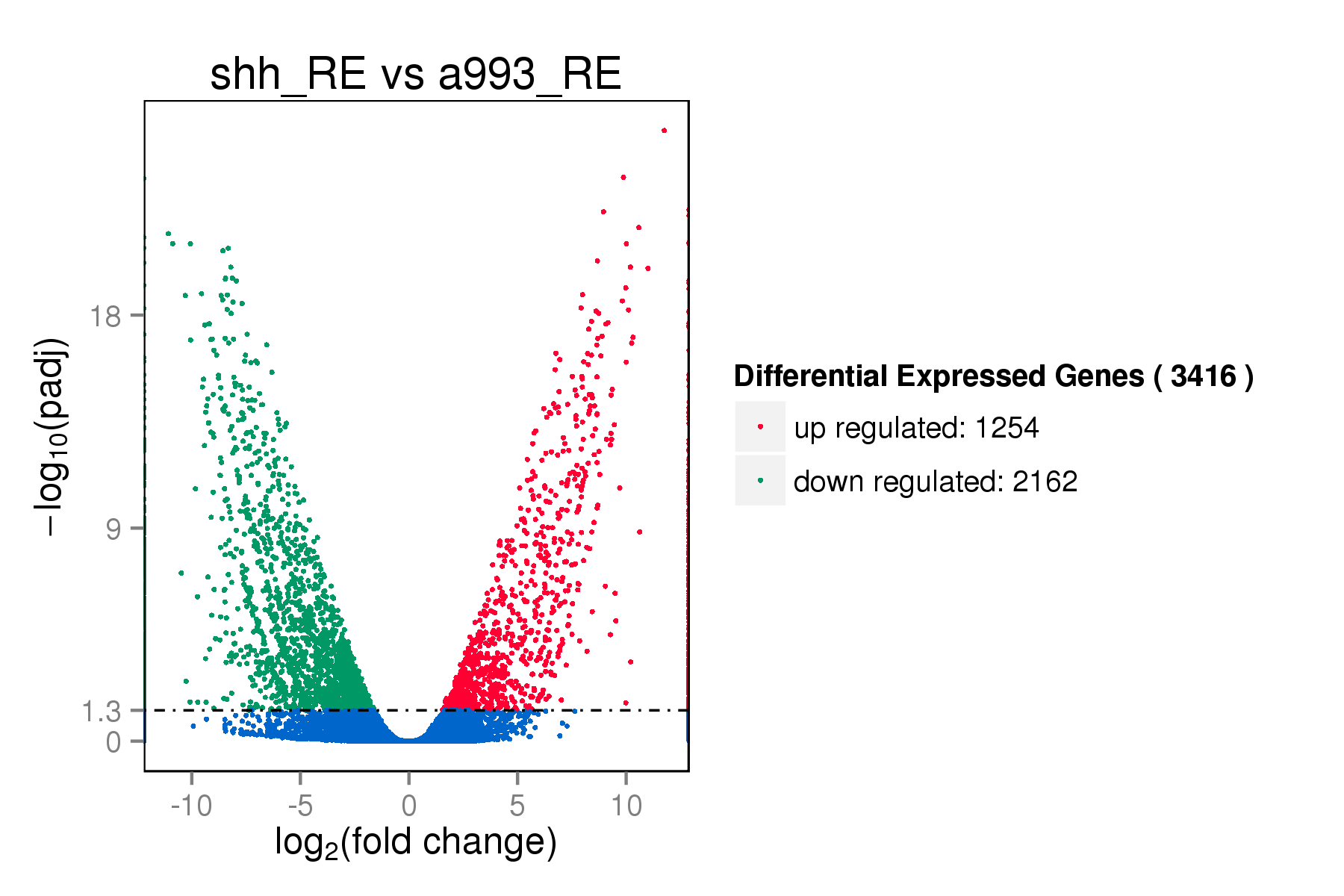
**

**Fig. S5** **Volcano plots of differentially expressed genes between root periderm of *S. miltiorrhiza* line shh and 99-3.** The mature root periderm of line shh was assigned as “shh_RE” and the mature root periderm of line 99-3 was assigned as “a993_RE”. Compared with 993, 1254 genes were upregulated and 2162 genes were downregulated in shh with a threshold padj < 0.05.

**
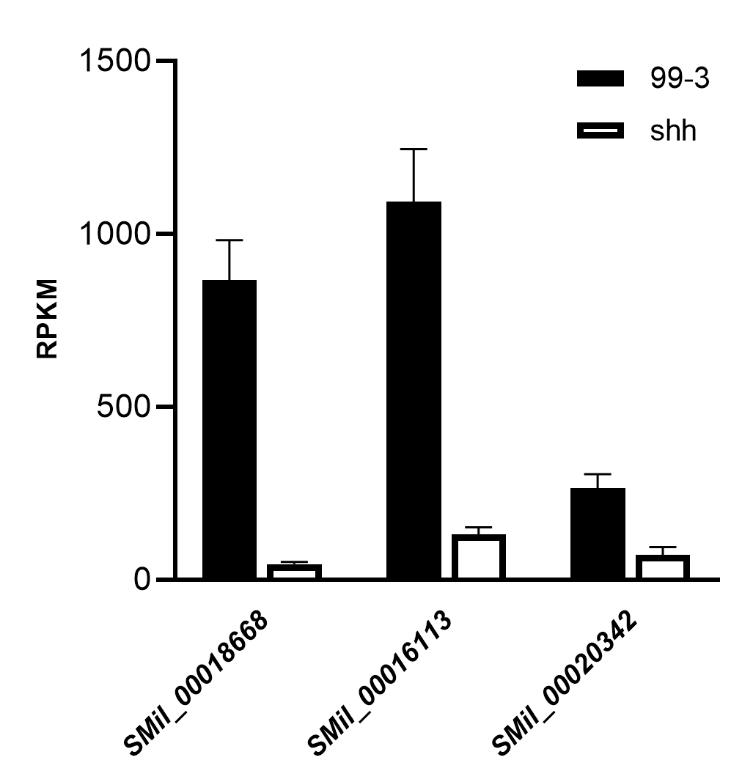
**

**Fig. S6** **The RPKM of *SMil_00016113*, *SMil_00018668*, and *SMil_00020342*** **in the mature periderm of 99-3 and shh, respectively.** *SMil_00016113*, *SMil_00018668*, and *SMil_00020342* showed the highest expression in mature periderm of 99-3 among 12 differentially expressed *Sm2OGDs*. Average RPKM values from three biological replicates are shown. Error bars represent standard deviations.

**
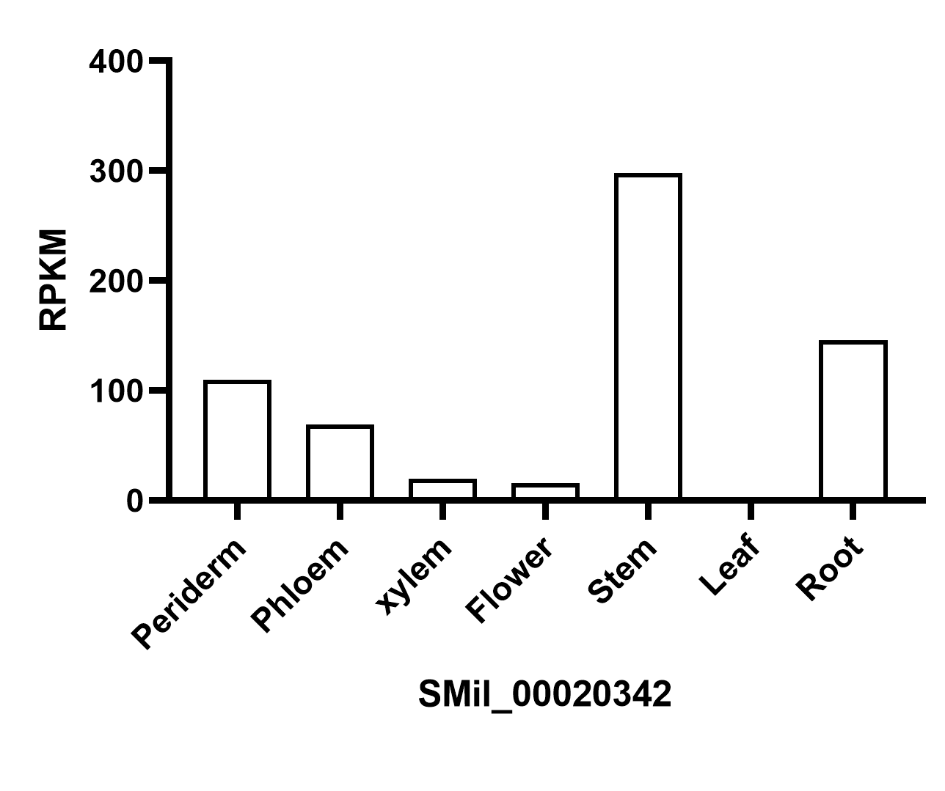
**

**Fig. S7** **The RPKM of *SMil_00020342*** **in different organs (leaf, root, stem, and flower) and root tissues (periderm, phloem, and xylem) of 99-3.** The expression level of SMil_00020342 in stem was highest. Data from published article (DOI: [10.1093/jxb/erx113](https://doi.org/10.1093/jxb/erx113)).

1.
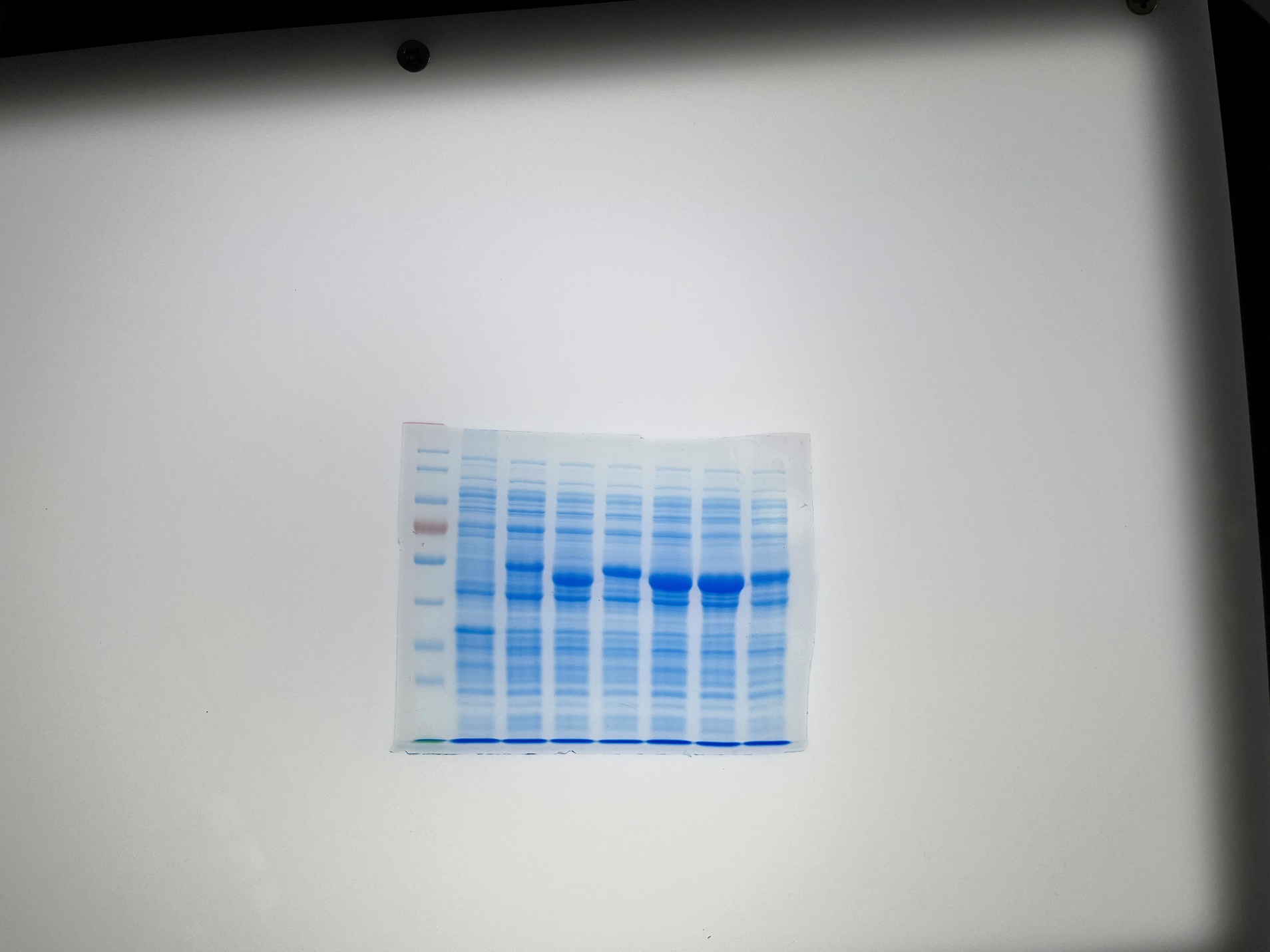

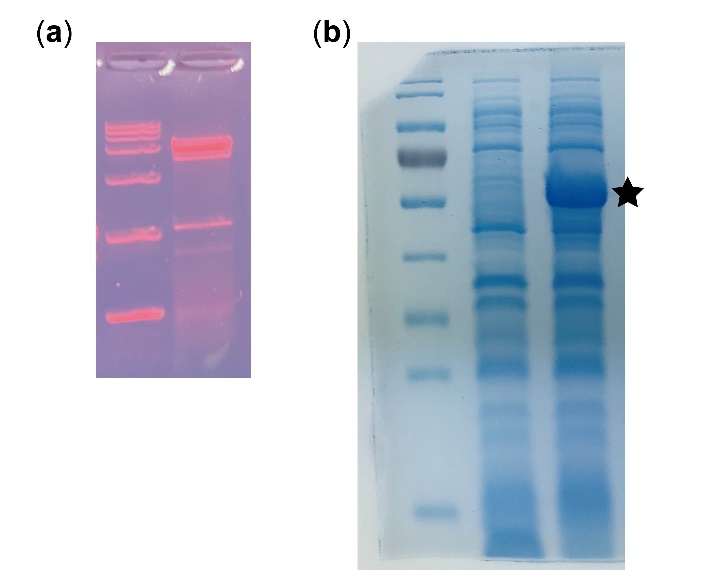
**
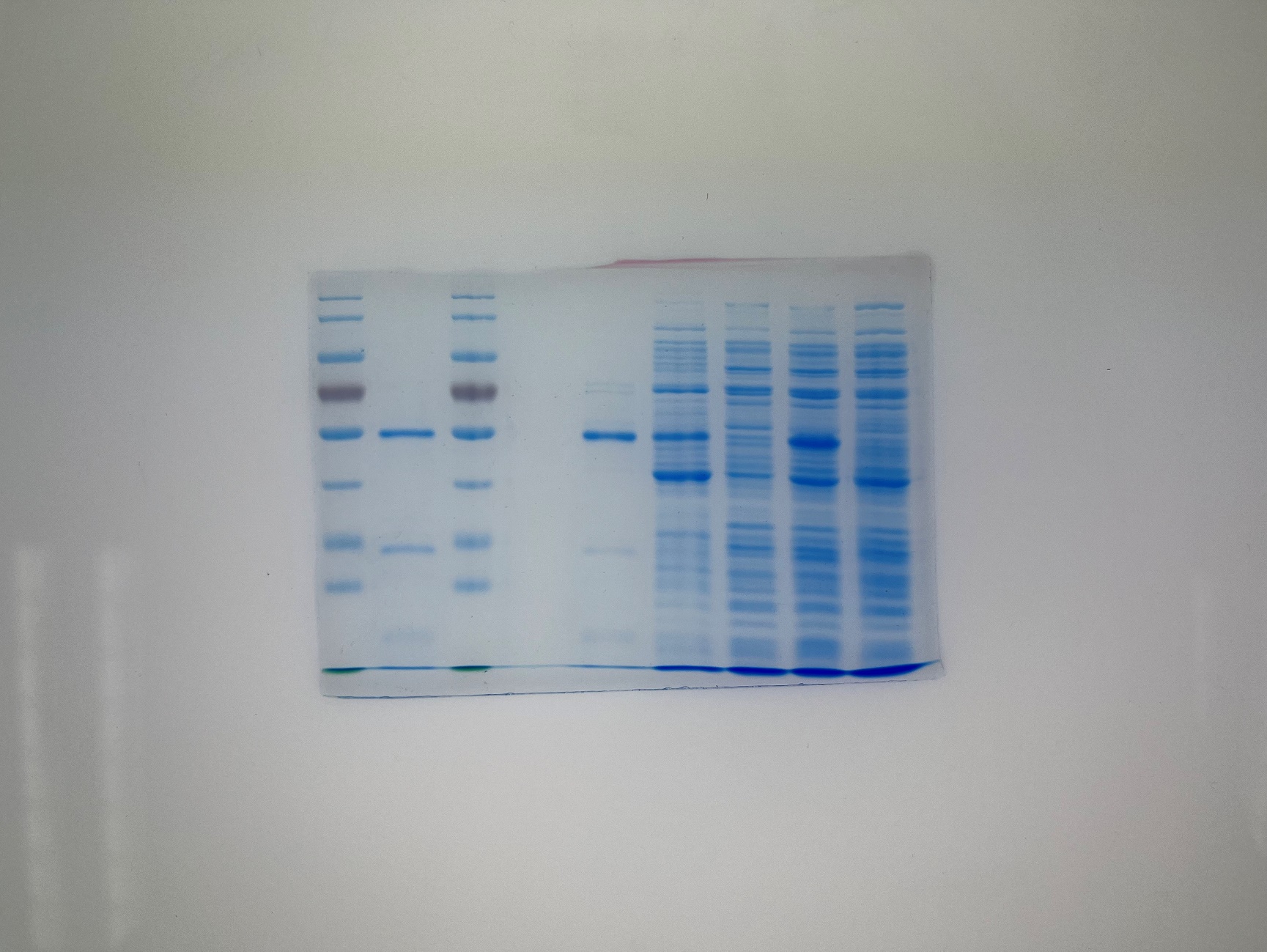
** **(b)**  **(c)**

**Fig. S8** **The** **electrophoresis diagram of recombinant vector pET-30a-Sm2OGD3 and Sm2OGD3 protein.** (a) The agarose gel electrophoresis diagram of recombinant vector pET-30a-Sm2OGD3 digested by BamHI and NotI. DNA marker 15000 shown in the first lane. (b) the SDS-PAGE electrophoresis diagram of 2OGD3 proteins. The first lane showed protein marker (10-180kD). The second to eighth lanes are shown pET-30a, pET-30a-Sm2OGD3_99-3, pET-30a-SmTIIAS, pET-30a-Sm2OGD3_DSS3, pET-30a-Sb2OGD3, pET-30a-Ss2OGD3_like, pET-30a-So2OGD3_like.The dashed box showed the soluble crude 2OGD3 proteins. (c) Purified Sm2OGD3_99-3 protein using Ni-NTA affinity chromatography and eluting with 300 mM imidazole.

**
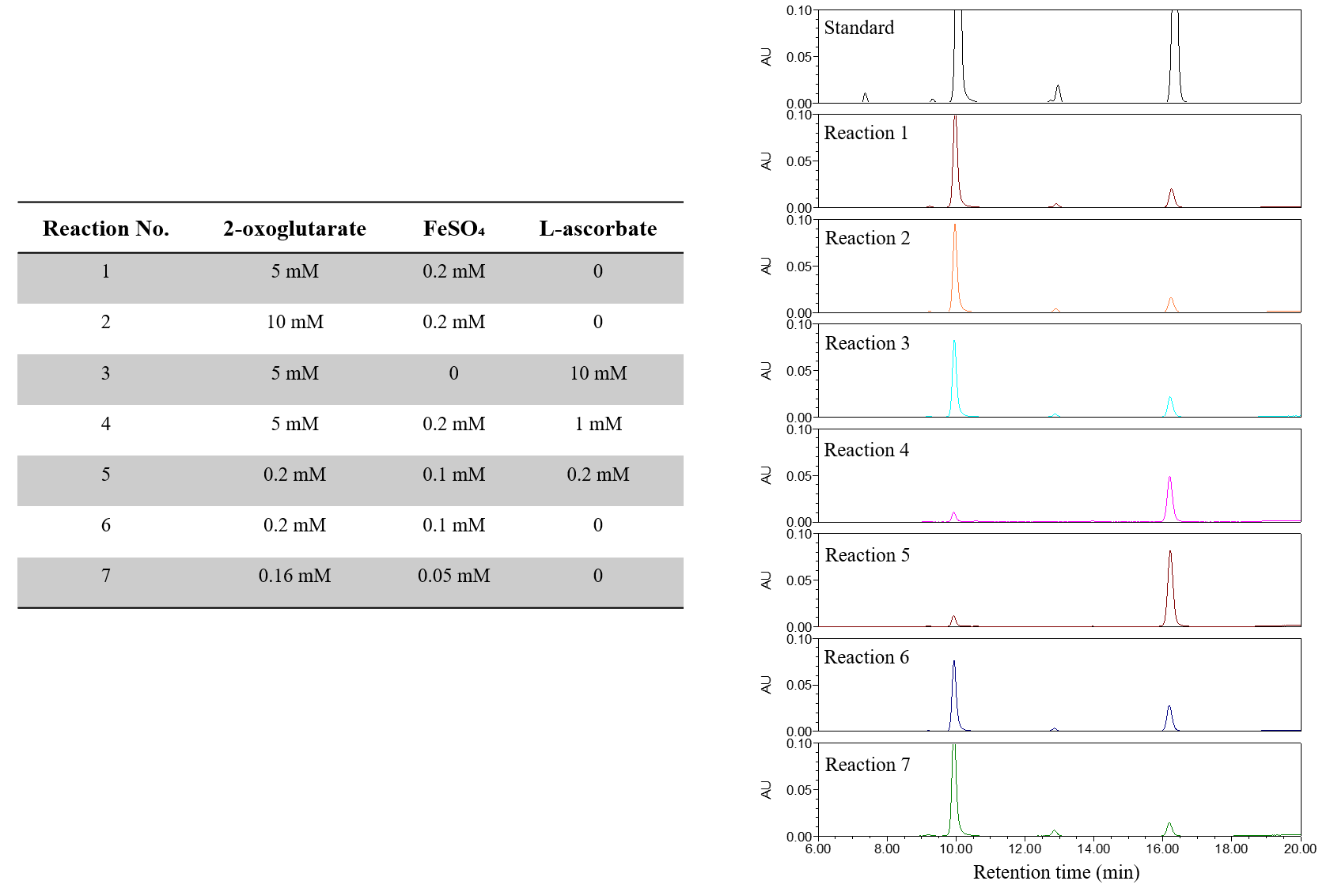
**

**Fig. S9** **The effect of the different components in the reaction system on the enzyme activity using 90** μ**M DHT as the substrate.**

**
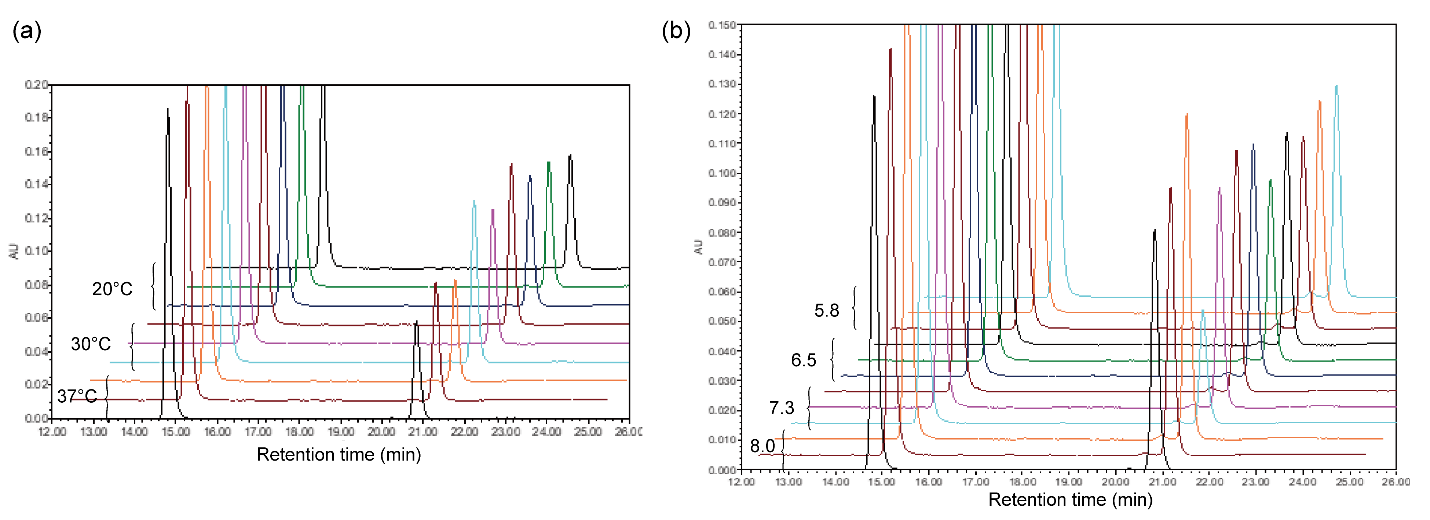
**

**Fig. S10**  **Effects of temperature and pH on enzyme activities of Sm2OGD3 using CT as substrate.** (a) The reactions at the different temperature of 20°C, 30°C and 37°C. (b) The pH gradient set to 5.8, 6.5, 7.3 and 8.0. All experiments were performed in triplicate.

**
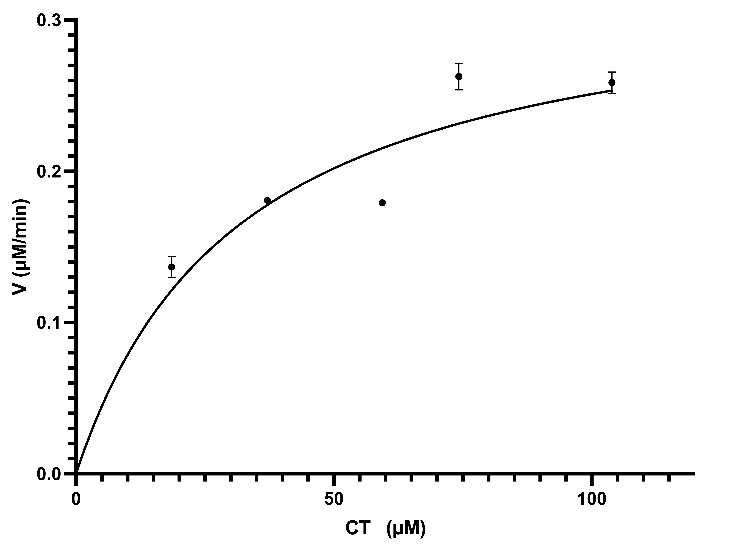

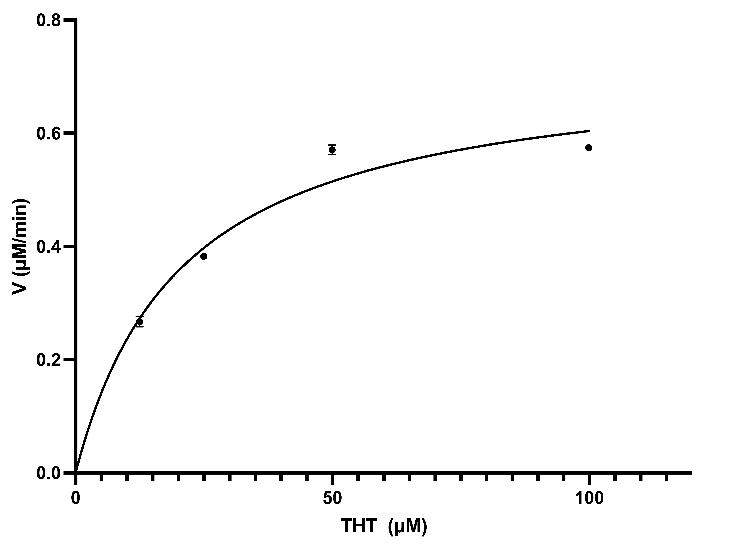

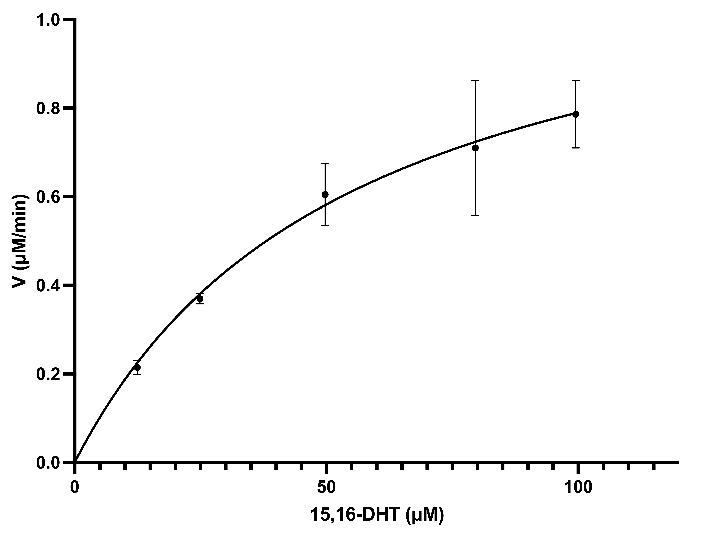
**

**Fig. S11** **Michaelis-Menten kinetic characterization of Sm2OGD3 with CT, THT, 15,16-DHT.** All experiments were performed in triplicate.

**
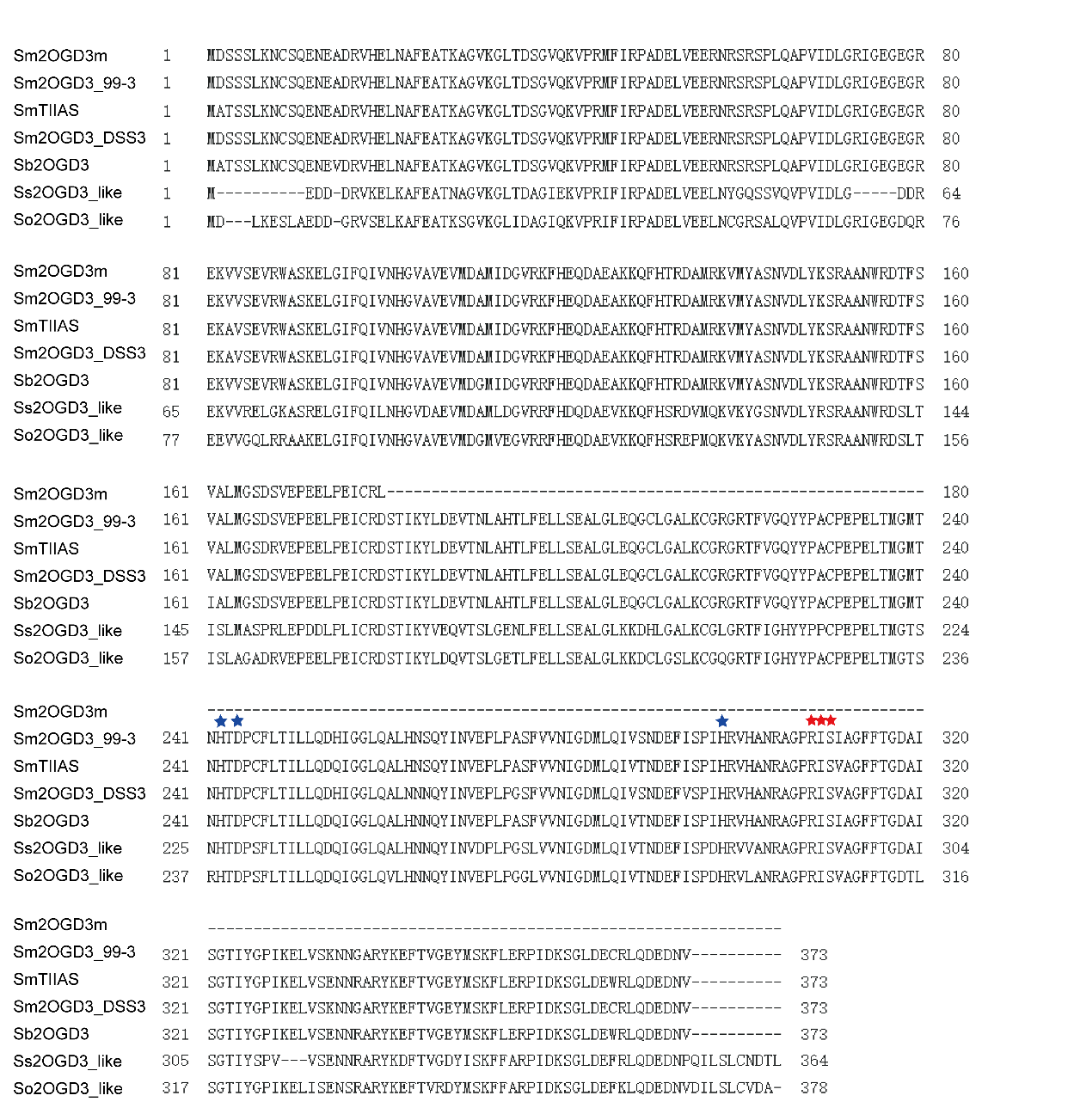
**

**Fig. S12** **Sequence alignments of orthologous genes of Sm2OGD3.** Blue asterisks indicate the 2-His-1-carboxylate facial triad (HX(D/E)X_50-210_H). Red asterisks indicate a conserved RXS motif with importance in 2OG binding. Sm, *Salvia miltiorrhiza*; Sb, *Salvia bowleyana*; Ss, *Salvia* *splendens*; So, *Salvia officinalis*. The protein sequences of SmTIIAS were down from NCBI under the accession numbers MW916096. The coding sequences of all orthologous genes of Sm2OGD3 were list in table S12.

**
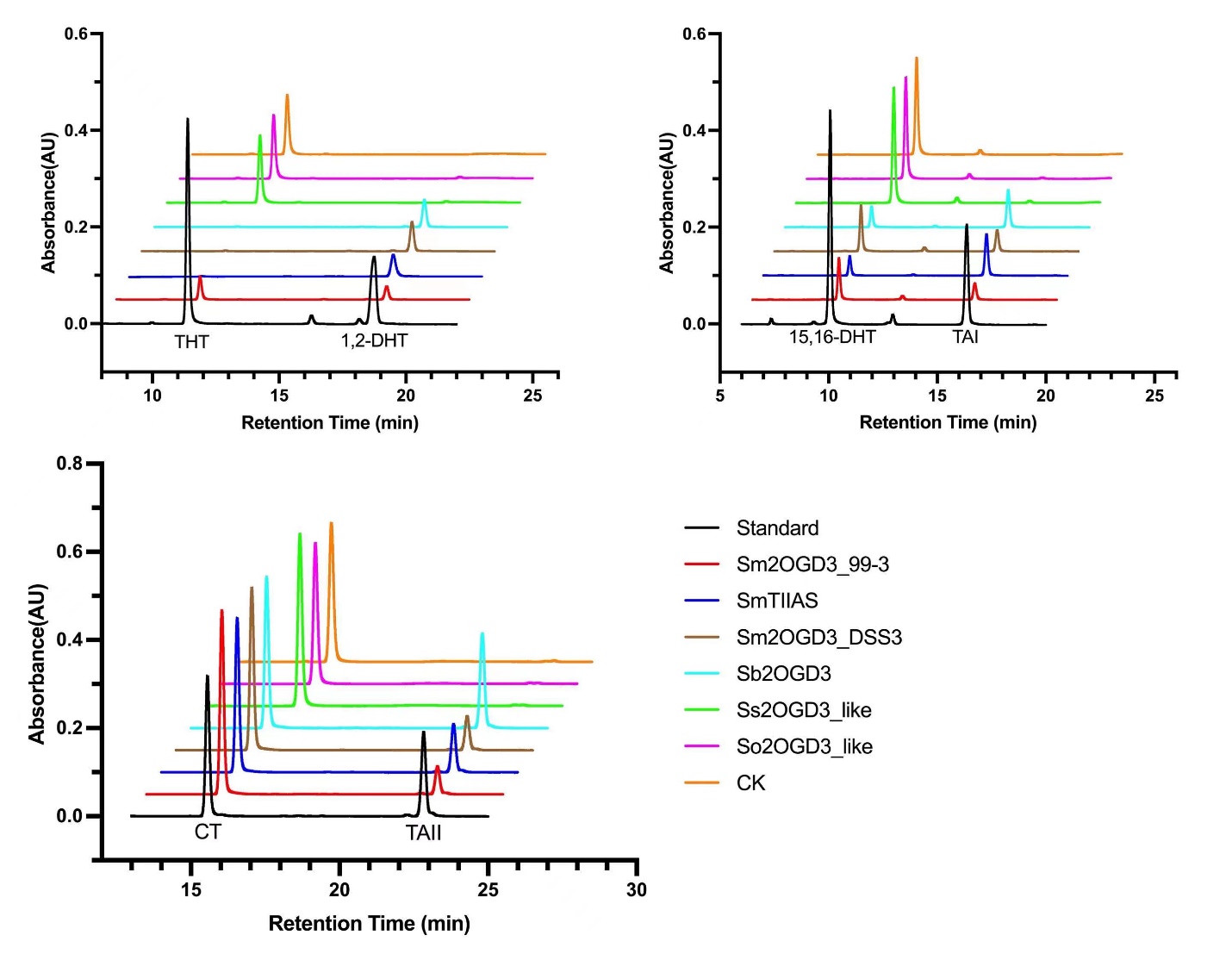
**

**Fig. S13** ***In vitro* enzyme activity assay of Sm2OGD3 orthologous proteins from *Salvia* genus.** Sm, *Salvia miltiorrhiza*; Sb, *Salvia bowleyana*; Ss, *Salvia* *splendens*; So, *Salvia officinalis*. Enzyme activity was analyzed *in vitro*. *E. coli* harboring the empty pET-30a vector was used as a control.

**
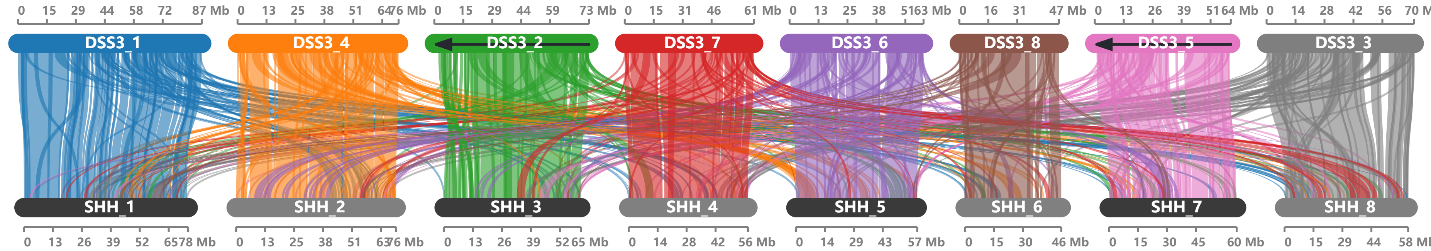
**

**
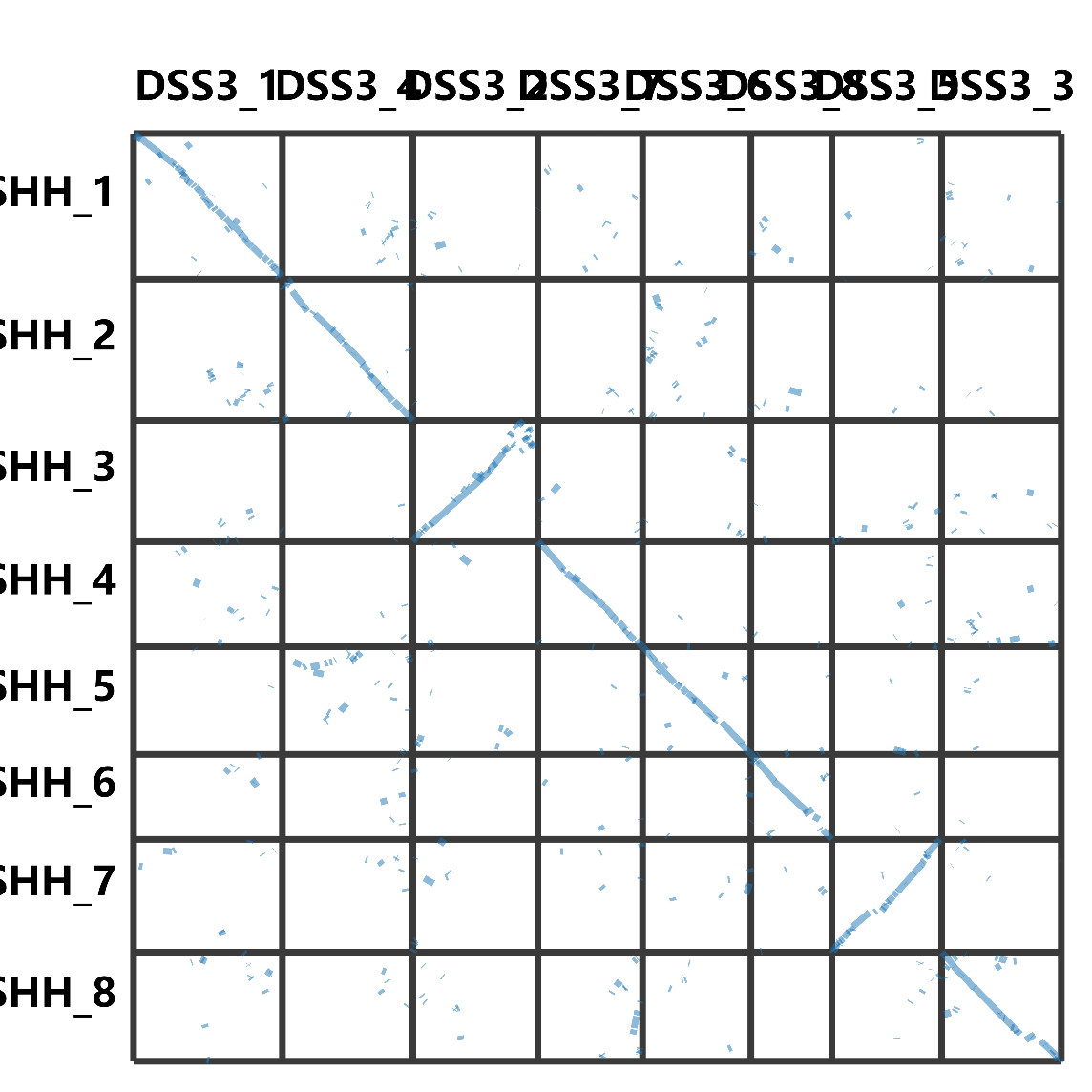
**

**Fig. S14** **Genomic collinearity analysis between shh assembly and DSS3 assembly.**

**Fig. S7** **Sequence alignments of Sm2OGD3, SmTIIAS and other 2OGDs from *Salvia* genus.** Blue asterisks indicate the 2-His-1-carboxylate facial triad (HX(D/E)X_50-210_H). Red asterisks indicate a conserved RXS motif with importance in 2OG binding. Green dots indicate the ten different amino acids between Sm2OGD3 and SmTIIAS. TIIAS, tanshinone IIA synthase; Sm, *Salvia miltiorrhiza*; Sbow, *Salvia bowleyana*; Smei, *Salvia meiliensis*; Stri, *Salvia trijuga*. The protein sequences of SmTIIAS, SbowTIIAS, SmeiTIIAS and StriTIIAS were down from NCBI under the accession numbers of MW916096, MW928604, MW928605 and MW928606, respectively.
